# Estimating the effects of hypothetical loneliness interventions on memory function among middle-aged and older adults in the United States

**DOI:** 10.1093/aje/kwag044

**Published:** 2026-03-09

**Authors:** Ryo Ikesu, Yingyan Wu, L Paloma Rojas-Saunero, Roch A Nianogo, Jacqueline M Torres, Ashwin Kotwal, Christina M Ramirez, Yusuke Tsugawa, Elizabeth Rose Mayeda

**Affiliations:** Department of Epidemiology, UCLA Fielding School of Public Health, Los Angeles, CA, United States; Division of General Internal Medicine and Health Services Research, David Geffen School of Medicine at UCLA, Los Angeles, CA, United States; Department of Health Promotion and Behavioral Sciences, Graduate School of Medicine, Kyoto University, Kyoto, Japan; Department of Epidemiology, UCLA Fielding School of Public Health, Los Angeles, CA, United States; Department of Epidemiology, UCLA Fielding School of Public Health, Los Angeles, CA, United States; Department of Epidemiology, UCLA Fielding School of Public Health, Los Angeles, CA, United States; Department of Epidemiology and Biostatistics, School of Medicine, University of California, San Francisco, San Francisco, CA, United States; Division of Geriatrics, Department of Medicine, University of California, San Francisco, San Francisco, CA, United States; Department of Biostatistics, UCLA Fielding School of Public Health, Los Angeles, CA, United States; Division of General Internal Medicine and Health Services Research, David Geffen School of Medicine at UCLA, Los Angeles, CA, United States; Department of Health Policy and Management, UCLA Fielding School of Public Health, Los Angeles, CA, United States; Department of Epidemiology, UCLA Fielding School of Public Health, Los Angeles, CA, United States

**Keywords:** loneliness, memory function, hypothetical intervention, time-varying analysis, targeted maximum likelihood estimation, Health and Retirement Study

## Abstract

Loneliness is associated with faster memory decline in mid- and late life, but it remains unclear whether interventions to ameliorate loneliness protect memory function. We examined the impact of sustained and one-time loneliness interventions on memory function among US middle-aged and older adults. Using the nationally representative Health and Retirement Study in 2006-2018, we estimated counterfactual average memory scores over 12 years of follow-up under the following scenarios: (A) a hypothetical intervention eliminating loneliness only at baseline (baseline intervention), (B) a hypothetical intervention eliminating loneliness for 10 years (sustained intervention), and (C) the natural course (no intervention). We used targeted maximum likelihood estimation to account for time-varying confounding. The analytic sample included 10 136 participants (median baseline age 64 years, representing 50 million community-dwelling adults). Over 12 years, estimated mean memory scores declined by 0.58 standardized units (95% CI, 0.56, 0.60) under the natural course; the difference in decline (vs natural course) was 0.00 standardized units (95% CI, −0.01, 0.01) under the baseline intervention and 0.02 standardized units (95% CI, −0.02, 0.05) under the sustained intervention. Compared to the natural course, we did not find evidence that either the baseline or sustained intervention was associated with better memory function over follow-up.

This article is linked to “Clarifying causal questions in population health research: anatomy of a causal estimand” (https://doi.org/10.1093/aje/kwag079).

## Introduction

Loneliness, a subjective and distressing experience of a discrepancy between one’s preferred interpersonal relationships and one’s actual interactions with others, has emerged as a pressing concern in society.^1-3^ Approximately 20%-40% of US older adults report feeling lonely.^4-9^ Studies have shown that older adults who feel lonely, particularly those with persistent loneliness,^10^ have lower memory function and faster memory decline,^10-13^ a hallmark of Alzheimer’s disease and related dementias. This growing body of evidence suggests that interventions to ameliorate loneliness, such as cognitive behavioral therapy targeting maladaptive social cognition (eg, negative thoughts about self-worth) or expanding opportunities for social connection, may help preserve late-life memory function and prevent dementia.^14-16^ The public health importance of identifying effective strategies to prevent dementia is underscored by its high burden: an estimated 7.2 million people in the United States are living with Alzheimer’s dementia, a number projected to increase to 13.9 million in 2060 as the population ages.^17^ Thus, effective strategies to slow memory decline among middle-aged and older adults are urgently needed.

While numerous studies have evaluated the associations of loneliness (lonely vs not lonely) with memory function and memory decline,^10-13^ no prior studies have estimated the effects of interventions to ameliorate loneliness compared with no intervention. Moreover, given that a prior study showed that persistent loneliness was associated with lower memory function and faster memory decline than transient loneliness,^10^ interventions may need to be sustained over time rather than delivered as a one-time intervention. A major barrier to evaluating the impact of sustained loneliness interventions involves the correct handling of time-varying confounders. Models of sustained loneliness are inherently models of time-varying loneliness measures; many confounders of the relationship between time-varying loneliness and cognition may also be time-varying. For instance, health-related behaviors (eg, physical activity and smoking) may influence future loneliness and memory function,^18-21^ but loneliness may affect future health-related behaviors.^22^^,^^23^ Memory function itself could also be a time-varying confounder, given the potential bidirectional relationship between loneliness and memory function (ie, potential reverse causation).^12^^,^^24^^,^^25^ As these time-varying confounders act not only as confounders but also as mediators between an exposure and an outcome, using traditional regression models to account for time-varying confounders would result in biased effect estimates. Advanced epidemiological methods, such as g-methods, can be used to account for such time-varying confounding^26-29^; however, they have been rarely used in the context of estimating the effects of loneliness on memory function.^10^

In this context, we examined the impacts of sustained and one-time hypothetical loneliness interventions on memory scores using nationally representative data for middle-aged and older adults in the United States. We estimated population-average memory scores under interventions that eliminated loneliness from the population and population-average memory scores under conditions that naturally occurred in the population (ie, the natural course).^30^ We hypothesized that a sustained intervention would be more effective than a one-time intervention. Under the consistency assumption that eliminating loneliness through any available means would have the same effect on memory function, we aimed to provide insight into the effects of population-level policies and programs targeting loneliness. We used the target trial emulation framework^31^^,^^32^ to facilitate causal inference using observational data and clarify the causal quantities of interest, and we applied targeted maximum likelihood estimation (TMLE) to account for potential time-varying confounding between time-varying loneliness and memory function.

## Methods

### Data and population

We used data from the Health and Retirement Study (HRS) from 2006 to 2018. HRS is a nationally representative longitudinal study of community-dwelling individuals over age 50 in the United States with a steady-state sample size of approximately 20 000 participants.^33^ Core interviews have been conducted every 2 years since 1992. To collect additional psychosocial and lifestyle information, the Psychosocial and Lifestyle Questionnaire (also referred to as the Leave-Behind Questionnaire) has been administered to a random half of HRS participants (ie, longitudinal data from the Leave-Behind Questionnaire are available at 4 year intervals) since 2006.^34^ Among 18 469 participants who were eligible for the 2006 core interview, we limited the sample to 17 105 participants aged 50 and older with non-zero sampling weights (ie, community-dwelling participants aged 50 and older) in that year. In the main analysis, we further limited the sample to 10 136 participants who were alive in 2018, regardless of their participation in the interviews during the study period (Figure S1). To account for the possibility of selection bias due to loss to follow-up, we repeated analyses with inverse probability weights for loss to follow-up after including participants who were lost to follow-up before 2018 (see *Sensitivity analyses*). We chose 2006 as the baseline year because some important covariates, such as social contact, first became available in the 2006 Leave-Behind Questionnaire. Missing values in the dataset were imputed with multiple imputation (see “Statistical analyses”).

HRS data collection was approved by the University of Michigan Institutional Review Board. All HRS participants provided informed consent. The proposed study used publicly available de-identified data and was exempt from review by the University of California, Los Angeles Institutional Review Board.

### Exposure variable

Loneliness was assessed every 2 years from 2006 to 2016, using the question at the biennial core interviews regarding loneliness over the week prior to the interview: “Do you feel lonely?” with response options yes/no. This one-item loneliness scale has a good discriminative ability for loneliness, compared to the gold-standard, Revised UCLA Loneliness Scale.^35^ As a sensitivity analysis, we repeated the analysis using a 3-item scale of the Revised UCLA Loneliness Scale,^36^ which was administered in the Leave-Behind Questionnaire every 4 years (see “Sensitivity analyses”). In our analysis, we assumed that the measured loneliness represented loneliness status for the entire years between study intervals (2 years for the one-item loneliness scale and 4 years for the Revised UCLA Loneliness Scale used for a sensitivity analysis; eg, for the one-item loneliness scale, we assume that loneliness status measured in 2006 represented loneliness status until 2008).

### Outcome variables

Our outcome was a composite memory score, assessed from 2008 to 2018, that combined direct and proxy memory assessments; it was developed to reduce potential bias due to missing direct memory scores due to cognitive impairment.^37^ At each biennial interview, participants’ memory was assessed with immediate and delayed recall of a 10-word list. For individuals too impaired to complete the interview, proxy informants, typically spouses, assessed the participants’ memory using a 5-point Likert scale and completed the 16-item version of the Jorm Informant Questionnaire for Cognitive Decline.^38^^,^^39^ The composite memory score was developed by calibrating those cognitive items against a comprehensive memory assessment with neuropsychological batteries in the Aging, Demographics, and Memory Study, a sub-study of HRS.^37^ Composite memory scores were standardized to the distribution of scores in the analytical sample at baseline for ease of interpretation, with lower scores reflecting lower memory function.

### Adjustment variables

We considered sociodemographic characteristics, health-related behaviors, and health conditions as potential confounders of the association between loneliness and memory scores. We considered age (in years) at baseline, sex/gender, race and ethnicity (non-Hispanic White, non-Hispanic Black, Hispanic, or other), educational attainment (less than high school, general educational development (GED), high school graduate, some college, or college graduate), birth in the southern US region (yes vs no) classified by the US Census region (Alabama, Arkansas, Delaware, Florida, Georgia, Kentucky, Louisiana, Maryland, Mississippi, North Carolina, Oklahoma, South Carolina, Tennessee, Texas, Virginia, Washington, DC, and West Virginia), and five personality traits (neuroticism, extraversion, openness to experience, agreeableness, and conscientiousness) based on 4-point scales^40^^,^^41^ as time-invariant covariates measured at baseline. See Supplementary material for justification regarding adjusting for birth in the southern US region and personality traits.

**Figure 1:**
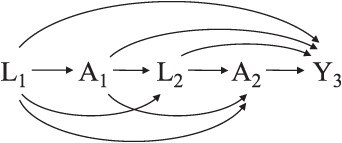
Causal diagram for loneliness and memory scores. For simplicity, this causal diagram shows the underlying causal structure to simulate memory scores at wave 3 (Y_3_). Similar causal structures were assumed for memory scores throughout the follow-up period (waves 2-7). L_1_ represents time-invariant covariates and time-varying covariates measured at baseline (wave 1). L_2_ represents time-varying covariates measured at wave 2. Time-invariant covariates measured at baseline included age at baseline, sex/gender, race and ethnicity, educational attainment, whether born in southern states, and personality traits (neuroticism, extraversion, openness to experience, agreeableness, and conscientiousness). Time-varying covariates included household wealth per person, employment status, social contact (marital status, participation in social activities, and contact with children, other family members, or friends), smoking status, frequency of vigorous physical activity, self-reported diagnosed comorbidities (diabetes, hypertension, and stroke), self-rated health, self-reported pain, depressive symptoms measured by CES-D score, activities of daily living limitations, and memory function in previous waves. A_1_ is a binary loneliness indicator at baseline and A_2_ is a subsequent loneliness status at wave 2. Y_3_ is a memory score at wave 3.

We treated all other variables as time-varying covariates, measured from 2006 to 2016, as they may have been influenced by past loneliness and could act as confounders of the relationship between current loneliness and future memory function (see causal diagram in Figure 1). Time-varying covariates included household wealth per person (defined as household wealth divided by the square root of the number of household members), employment status (currently working vs not), and social contact. Social contact was assessed using five items (yes vs no): married or partnered, at least monthly participation in social activities (sport or social groups, religious groups, education or training courses, or meetings), and at least monthly contact (including face to face, telephone, or written/e-mail) with each of children, other family members, and friends.^42^ Health-related behaviors included smoking status (current smoker vs not) and frequency of vigorous physical activity (at least once per month vs not). Health conditions included self-reported diagnosed comorbidities (diabetes, hypertension, and stroke), self-rated health (excellent/very good, good, or fair/poor), self-reported pain (moderate/severe pain vs not),^43-45^ depressive symptoms (Center for Epidemiological Studies-Depression [CES-D] score excluding the loneliness item [score range: 0-7]),^46^ and activities of daily living limitations (any difficulty in dressing, eating, bathing and showering, walking across a room, and getting in and out of bed; score range 0-6). These health-related behaviors and health conditions were included because they may cause both loneliness and memory decline.^47-49^ Notably, to account for the potential reverse causation between loneliness and memory function, memory scores were treated not only as the outcome variable but also as a time-varying confounder^12^^,^^24^^,^^25^ (see Supplementary material for details).

### Statistical analysis

First, we examined the trajectories of loneliness biennially from 2006 to 2016 (six evaluations of loneliness for each participant). To assess the extent of within-person variability in loneliness, we calculated the proportions of the following three categories among participants with loneliness measurements at all six waves (2006-2016): (1) those who remained lonely throughout follow-up, (2) those who never experienced loneliness throughout follow-up, and (3) those whose loneliness status varied throughout follow-up. We focused on participants with loneliness measurements at all six waves for this descriptive analysis because imputed values of loneliness can vary among multiple imputed datasets (see multiple imputation description below).

Next, to evaluate the effects of hypothetical loneliness interventions on memory function, we emulated a randomized trial (target trial emulation framework).^31^^,^^32^ Our approach entailed (1) conceptualizing the target trial and (2) implementing TMLE to estimate the effects of the desired treatment scenarios compared to the natural course (no intervention), accounting for baseline and time-varying confounding.

We conceptualized a target trial where eligible study participants were defined as community-dwelling individuals aged 50 and older in the United States. Then, we estimated counterfactual average memory scores over follow-up under the following three scenarios: (A) a hypothetical intervention eliminating loneliness at baseline but not intervening at later time points (baseline loneliness intervention), (B) a hypothetical intervention eliminating loneliness at each time point (sustained loneliness intervention), and (C) the natural course (ie, no intervention).

To assess the effectiveness of the baseline and sustained loneliness interventions, we compared counterfactual memory scores at each study wave over 12 years after baseline under the baseline and sustained interventions to the natural course (see Supplementary material for causal estimands). We focused on a per-protocol effect in our target trial emulation because we sought to estimate the effects of eliminating loneliness from the population. Detailed specifications of the target trial are shown in Table 1.

**Table 1:** Specification and emulation of a target trial that evaluates the effect of loneliness interventions on memory scores in the community-dwelling US population aged 50 years and older.

**Trial component**	**Target trial specification**	**Emulation using HRS data**
Eligibility criteria	Community-dwelling individuals in the United States aged 50 years and older at baseline	Community-dwelling individuals aged 50 years and older who participated in the 2006 HRS interview and remained in the study through 2018^a^
Scenarios of interest	i. Baseline loneliness intervention^b^ ii. Sustained loneliness intervention^c^ iii. Natural course (no intervention on loneliness)	Scenarios of interest were the same as the target trial specification. Loneliness was measured biennially with the question “Do you feel lonely?” (yes/no response)
Assignment procedures	Individuals were randomly assigned to one of the three interventions of interest; they were not blinded to the assignment	All individuals in the sample were assigned to each of three interventions of interest
Follow-up period	From 2006 to 2018, biennially	Same as the target trial specification
Outcome	Memory score	Same as the target trial specification
Causal contrast of interest	Per-protocol effect^d^	Same as the target trial specification
Analysis plan	TMLE to account for time-varying confounding	Same as the target trial specification

Abbreviations: HRS, Health and Retirement Study; TMLE, targeted maximum likelihood estimation.

a In a sensitivity analysis, all community-dwelling individuals aged 50 years and older who participated in the 2006 HRS interview were included in the sample.

b Interventions to eliminate loneliness only at baseline in the population.

c Interventions to eliminate loneliness throughout follow-up in the population.

d Effect of following the intervention of interest, as opposed to being assigned to the intervention.

We estimated the effects of the loneliness interventions while accounting for baseline and time-varying confounders using TMLE. TMLE provides valid effect estimates under several assumptions, including conditional exchangeability, consistency, and positivity (see Supplementary material for details). Compared to other epidemiological methods that account for time-varying confounding (eg, the g-formula), TMLE is robust to model misspecification in estimating causal effects as it yields consistent estimates of causal effects as long as at least one of exposure or outcome is modeled correctly (doubly robust property).^50-52^ Briefly, TMLE first estimates an outcome conditional on covariates and the exposure. Then, the outcome is updated with an estimated exposure conditional on covariates. Finally, the updated outcome is used to simulate counterfactual outcomes in the population. To reduce concerns of potential model misspecification for the exposure and outcome, we applied TMLE using the R package lmtp^53^ with Super Learner (R package SuperLearner).^50-52^ Super Learner is an ensemble method that combines different machine learning algorithms (base learners) to enhance predictive performance^54^^,^^55^ and thus mitigates the concern of model misspecification with its flexibility. In this analysis, generalized linear models, random forest (R package randomForest), and multivariate adaptive regression splines (R package earth) were used as base learners with 10-fold cross-validation. Applying TMLE with the variables mentioned above, we simulated average memory scores in the population at each biennial wave (7 waves from 2006 to 2018) under the three scenarios. The outcome (memory score) at each wave was modeled using the exposure and covariates measured at all previous waves, without including variables measured at the concurrent wave (Figure 1). The exposure (loneliness) at each wave was modeled using the exposure from all previous waves and covariates (including memory scores) from all previous waves and the concurrent wave. In contrast to the analysis for the sustained loneliness intervention, the analysis for the baseline loneliness intervention did not require adjustment for time-varying confounding. We estimated memory score change at each study wave under each scenario by subtracting the mean baseline memory score from the estimated mean memory score at each follow-up wave. We then compared the memory score change between scenarios by taking the difference in the estimated mean memory score change at each wave between scenarios.

To explore heterogeneity in the effects of the loneliness interventions, we repeated analyses stratified by baseline age (age < 65 years, age $\ge$65 years), sex/gender (women, men), and social isolation status (yes/no) at baseline. Based on a previous study,^42^ participants were considered socially isolated if they met at least two of the following five conditions: not married or partnered, less than monthly participation in social activities, and less than monthly contact with children, other family members, or friends.

We applied multilevel multiple imputation (5 imputed datasets) to handle missing data in longitudinal data, using the fully conditional specification approach using the R package mice^56-58^ (see Supplementary material and Table S1 for details). To obtain unbiased estimates with multiple imputation, the probability of missingness needs to be at random, conditional on observed variables (ie, the missing at random assumption). Percent missingness prior to imputation ranged from 0% to 59% across variables (Table 2 and Table S2). The percentage of missing values for loneliness and memory scores in the main analytic sample is presented in Table S3. Variables for contact with children/other family members/friends were available in the Leave-Behind Questionnaire, which was administered to a rotating random half of participants every 4 years. Therefore, by design, approximately 50% of participants had missing values for these items at baseline.

**Table 2:** Baseline characteristics of the sample, stratified by baseline loneliness status.

	**Total**	**Baseline loneliness**		
		**No**	**Yes**	**Missing**
Unweighted *N*	10 136	8293	1461	382
Weighted *N*^a^	50 209 090	41 277 864	7 024 598	1 906 628
Age (median [Q1, Q3])	64 [58, 70]	65 [58, 70]	64 [57, 70]	64 [58, 69]
Missing	0.00%	0.00%	0.00%	0.00%
Women (%)	6140 (60.6)	4996 (60.2)	1044 (71.5)	100 (26.2)
Missing	0.00%	0.00%	0.00%	0.00%
Race and ethnicity (%)				
Non-Hispanic White	7443 (73.4)	6340 (76.5)	861 (58.9)	242 (63.4)
Non-Hispanic Black	1376 (13.6)	1038 (12.5)	285 (19.5)	53 (13.9)
Hispanic	1048 (10.3)	708 (8.5)	273 (18.7)	67 (17.5)
Other	269 (2.7)	207 (2.5)	42 (2.9)	20 (5.2)
Missing	0.00%	0.00%	0.00%	0.00%
Educational attainment (%)				
Less than high school degree	1751 (17.3)	1210 (14.6)	422 (28.9)	119 (31.2)
GED	453 (4.5)	357 (4.3)	83 (5.7)	13 (3.4)
High school graduate	3118 (30.8)	2573 (31.0)	429 (29.4)	116 (30.4)
Some college	2353 (23.2)	1971 (23.8)	321 (22.0)	61 (16.0)
College graduate	2459 (24.3)	2180 (26.3)	206 (14.1)	73 (19.1)
Missing	0.02%	0.02%	0.00%	0.00%
Birth in the southern US region (%)^b^	3253 (32.1)	2587 (31.2)	520 (35.6)	146 (38.4)
Missing	0.10%	0.07%	0.14%	0.52%
Measures of personality^c^				
Neuroticism (median [Q1, Q3])	3 [2, 4]	3 [3, 4]	2 [2, 3]	3 [2, 3]
Missing	15.49%	12.89%	19.51%	56.54%
Extraversion (median [Q1, Q3])	2 [1, 2]	2 [1, 2]	2 [1, 2]	2 [1, 2]
Missing	15.46%	12.83%	19.64%	56.54%
Openness to experience (median [Q1, Q3])	2 [2, 2]	2 [2, 2]	2 [2, 2]	2 [2, 3]
Missing	15.84%	13.17%	20.26%	57.07%
Agreeableness (median [Q1, Q3])	1 [1, 2]	1 [1, 2]	1 [1, 2]	2 [1, 2]
Missing	15.48%	12.84%	19.71%	56.54%
Conscientiousness (median [Q1, Q3])	2 [1, 2]	2 [1, 2]	2 [1, 2]	2 [1, 2]
Missing	15.56%	12.90%	19.85%	56.81%
Household wealth per person (median [Q1, Q3])	171 120 [47 343, 439 447]	191 161 [60 104, 480 000]	77 423 [6010, 250 000]	147 078 [48 028, 357 018]
Missing	0.00%	0.00%	0.00%	0.00%
Currently working (%)	4773 (47.1)	4062 (49.0)	494 (33.8)	217 (57.0)
Missing	0.06%	0.06%	0.00%	0.26%
Measures of social contact				
Married or partnered (%)	7190 (70.9)	6228 (75.1)	619 (42.4)	343 (89.8)
Missing	0.02%	0.01%	0.07%	0.00%
Participation in social activities (monthly) (%)	6447 (83.6)	5374 (83.6)	886 (82.0)	187 (91.7)
Missing	23.90%	22.48%	26.01%	46.60%
Contact with children (monthly) (%)	3831 (87.6)	3293 (87.7)	494 (87.0)	44 (88.0)
Missing	56.86%	54.72%	61.12%	86.91%
Contact with other family members (monthly) (%)	3462 (78.7)	2976 (78.8)	449 (78.6)	37 (72.5)
Missing	56.62%	54.48%	60.92%	86.65%
Contact with friends (monthly) (%)	3752 (84.8)	3264 (86.0)	451 (77.6)	37 (75.5)
Missing	56.32%	54.21%	60.23%	87.17%
Socially isolated (%)^d^	1168 (27.8)	937 (25.9)	219 (41.2)	12 (25.0)
Missing	58.52%	56.30%	63.59%	87.43%
Current smoking (%)	1226 (12.2)	931 (11.3)	246 (16.9)	49 (13.2)
Missing	0.75%	0.68%	0.55%	3.14%
Vigorous physical activity at least once per month (%)	4540 (44.8)	3907 (47.2)	469 (32.1)	164 (43.0)
Missing	0.12%	0.11%	0.14%	0.26%
Self-reported diagnosed comorbidities (%)				
Diabetes	1563 (15.4)	1176 (14.2)	319 (21.8)	68 (17.8)
Missing	0.00%	0.00%	0.00%	0.00%
Hypertension	5095 (50.3)	4061 (49.0)	828 (56.7)	206 (53.9)
Missing	0.00%	0.00%	0.00%	0.00%
Stroke	418 (4.1)	297 (3.6)	94 (6.4)	27 (7.1)
Missing	0.00%	0.00%	0.00%	0.00%
Self-rated health (%)				
Excellent/very good	4946 (48.8)	4374 (52.8)	417 (28.6)	155 (40.6)
Good	3128 (30.9)	2576 (31.1)	418 (28.7)	134 (35.1)
Fair/poor	2054 (20.3)	1338 (16.1)	623 (42.7)	93 (24.3)
Missing	0.08%	0.06%	0.21%	0.00%
Moderate or severe pain (%)	2214 (21.9)	1560 (18.8)	552 (37.8)	102 (27.0)
Missing	0.14%	0.10%	0.14%	1.05%
CES-D score without loneliness item (median [Q1, Q3])^e^	0 [0, 2]	0 [0, 1]	3 [1, 5]	2 [1, 5]
Missing	4.26%	0.39%	1.51%	98.95%
ADL score (median [Q1, Q3])^f^	0 [0, 0]	0 [0, 0]	0 [0, 1]	0 [0, 0]
Missing	0.03%	0.01%	0.00%	0.52%

Abbreviations: ADL, activities of daily living; CES-D, Center for Epidemiological Studies-Depression; GED, general educational development; HRS, Health and Retirement Study; Q1, first quartile; Q3, third quartile. Values in this table were based on unweighted samples. Percentages for categorical values were calculated among non-missing values for each characteristic.

a Weighted numbers of Health and Retirement Study participants were calculated with sampling weights for community-dwelling adults in 2006.

b Birth in the southern US region was based on self-reported state of birth classified by US Census region, including Alabama, Arkansas, Delaware, Florida, Georgia, Kentucky, Louisiana, Maryland, Mississippi, North Carolina, Oklahoma, South Carolina, Tennessee, Texas, Virginia, Washington, D.C., and West Virginia.

c Measures of personality (neuroticism, extraversion, openness to experience, agreeableness, and conscientiousness) were scored based on 4-point scales ranging from 1 to 4 with higher scores more consistent with the corresponding trait.

d Participants were considered socially isolated if they met at least two of the following five conditions: not married or partnered, less than monthly participation in social activities, and less than monthly contact with children, other family members, or friends.

e CES-D score without loneliness item; score range 0-7.

f ADL score was defined by assigning one point to each of any difficulty in dressing, eating, bathing and showering, walking across a room, and getting in and out of bed; score range 0-6.

To obtain nationally representative estimates, analyses incorporated sampling weights for community-dwelling individuals in 2006 provided by HRS. Statistical analyses were conducted using R version 4.1.3. Dataset construction and analysis code is available at: https://github.com/Mayeda-Research-Group/hypothetical_loneliness_intervention.

### Sensitivity analyses

We performed sensitivity analyses. First, to assess whether the results were sensitive to a different measure of loneliness, we repeated the analysis using a 3-item scale of the Revised UCLA Loneliness Scale, which was administered in the Leave-Behind Questionnaire every 4 years.^36^ Participants were asked about how much they felt (1) they lacked companionship, (2) they were left out, and (3) they were isolated from others on a 3-point scale (“often,” “some of the time,” or “hardly ever or never”). We classified participants as lonely if they responded “often” or “some of the time” to at least one of the three questions and not lonely if they responded “hardly ever or never” to all three questions, based on previous studies.^6^^,^^44^ As a rotating random half of the core survey participants were asked to complete the Leave-Behind Questionnaire (ie, longitudinal data are available at 4 year intervals), the interval between observations for this sensitivity analysis was 4 years.^34^ In this sensitivity analysis, the baseline year was 2006 for participants eligible for the Leave-Behind Questionnaire in 2006, and it was 2008 for those eligible in 2008 (*n* = 16 977). We additionally adjusted for baseline year in this analysis to account for this difference. Second, to account for the possibility that those who felt lonely were more likely to become lost to follow-up than others (selection bias due to loss to follow-up), we estimated inverse probability weights for loss to follow-up (including both death and drop out) at each wave using the covariates mentioned above and repeated the analysis applying the product of the inverse probability weights and sampling weights in the full sample (*n* = 17 105). In doing so, the effect of the hypothetical interventions was estimated under the assumption that loss to follow-up could have been prevented over follow-up. Third, to assess the sensitivity of our results to the use of sampling weights, we repeated the main analysis without sampling weights. Fourth, to address the possibility that the outcome was correlated within a household, we conducted a sensitivity analysis after limiting the sample to only one participant within a household (we included the participant with the smallest person identifier number within a household). Fifth, we repeated the main analysis by increasing the number of imputed datasets from five to ten. Finally, to assess potential model misspecification in our TMLE analysis, we compared the observed mean memory score change over follow-up and the memory score change estimated from TMLE under the natural course. A close match between these two quantities would mitigate concerns of potential model misspecification.^30^

## Results

Among 10 136 participants (median age 64, representing 50 209 090 community-dwelling adults) in the main analytic sample, 14.4% experienced loneliness at baseline (unweighted in Table 2, applying sampling weights in Table S4, after applying multiple imputation in Table S5). The percentage of women, racially and ethnically minoritized people, and people without a high school degree was higher among those who were lonely vs not lonely at baseline. Those who were lonely at baseline tended to possess lower per-person household wealth, were less likely to be working, were less socially connected, and had poorer health and health-related behaviors than those who were not lonely at baseline.

**Figure 2:**
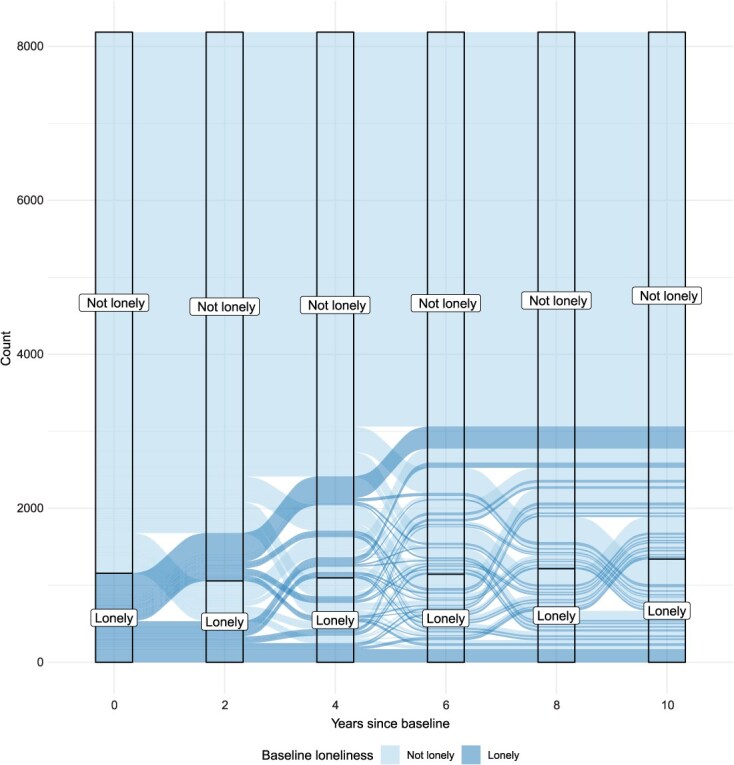
Prevalence of loneliness in the sample during follow-up. This figure is based on 8184 Health and Retirement Study participants (representing 39 892 097 community-dwelling US adults) with loneliness assessed at all six waves (2006-2016).

Among 8184 participants with loneliness assessed at all six waves (80.7% of the analytic sample), 169 (2.1%) experienced persistent loneliness (lonely at all waves), 5122 (62.6%) experienced persistent non-loneliness (never lonely), and 2893 (35.3%) experienced transient loneliness (variability in loneliness across waves) (Figure 2 and Table S6).

**Figure 3:**
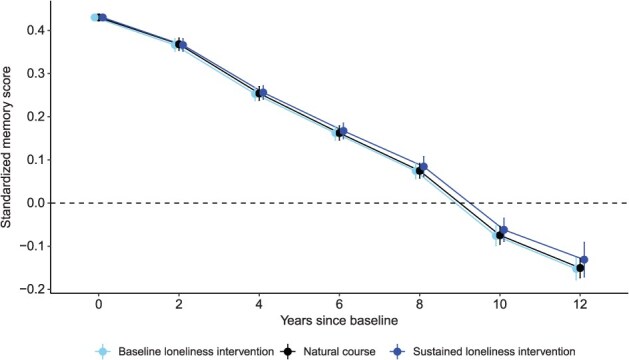
Estimated mean (95% CI) memory score trajectories under hypothetical loneliness interventions. Estimates were obtained adjusting for baseline covariates (age at baseline, sex/gender, race and ethnicity, educational attainment, birth in the southern US region, and personality traits), time-varying covariates (household wealth per person, employment status, social contact [marital status, participation in social activities, and contact with children, other family members, or friends], smoking status, frequency of vigorous physical activity, self-reported diagnosed comorbidities [diabetes, hypertension, and stroke], self-rated health, self-reported pain, depressive symptoms measured by CES-D score, and activities of daily living limitations), and memory function in previous waves.

In the TMLE analysis, compared to the natural course, we did not find evidence that the sustained loneliness intervention was associated with better memory scores over 12 years (Table 3 and Figure 3). Over 12 years of follow-up, estimated mean memory scores declined by 0.58 standardized units (95% CI, 0.56 to 0.60) under the natural course and 0.56 standardized units (95% CI, 0.52 to 0.60) under the sustained intervention. The estimated difference in decline was 0.02 standardized units (95% CI, −0.02 to 0.05), corresponding to a 3.3% reduction in memory decline at the population level (calculated as the estimated percentage difference in mean memory score decline divided by the estimated mean memory score decline under the natural course). The baseline loneliness intervention was also not associated with better memory function throughout follow-up compared to the natural course (Table 3 and Figure 3).

**Table 3:** Estimated mean change in memory scores from baseline under natural course and hypothetical loneliness interventions.

**Years since baseline**	**Estimated mean memory change from baseline**			**Estimated impact of interventions**			
	**Natural course [A] (95% CI)**	**Baseline loneliness intervention [B] (95% CI)**	**Sustained loneliness intervention [C] (95% CI)**	**Baseline loneliness intervention vs natural course**		**Sustained loneliness intervention vs natural course**	
				**Difference [B]—[A] (95% CI)**	Percentage reduction 100* ([A]—[B])/[A]	**Difference [C]—[A] (95% CI)**	Percentage reduction 100* ([A]—[C])/[A]
2 years	−0.06 (−0.08 to −0.05)	−0.06 (−0.08 to −0.05)	NA	0.00 (−0.01 to 0.00)	−3.1	NA	NA
4 years	−0.18 (−0.19 to −0.16)	−0.18 (−0.19 to −0.16)	−0.17 (−0.19 to −0.16)	0.00 (−0.01 to 0.00)	−0.8	0.00 (−0.01 to 0.01)	1.2
6 years	−0.27 (−0.28 to −0.25)	−0.27 (−0.29 to −0.25)	−0.26 (−0.28 to −0.24)	0.00 (−0.01 to 0.01)	−0.1	0.00 (−0.01 to 0.02)	1.9
8 years	−0.36 (−0.37 to −0.34)	−0.36 (−0.37 to −0.34)	−0.35 (−0.37 to −0.32)	0.00 (−0.01 to 0.01)	−0.1	0.01 (−0.01 to 0.03)	2.8
10 years	−0.50 (−0.53 to −0.48)	−0.51 (−0.53 to −0.48)	−0.49 (−0.52 to −0.46)	0.00 (−0.01 to 0.01)	−0.2	0.01 (−0.01 to 0.03)	2.5
12 years	−0.58 (−0.60 to −0.56)	−0.58 (−0.61 to −0.56)	−0.56 (−0.60 to −0.52)	0.00 (−0.01 to 0.01)	−0.4	0.02 (−0.02 to 0.05)	3.3

Abbreviations: CES-D, Center for Epidemiological Studies-Depression; NA, not applicable.

Estimated mean memory change from baseline was calculated by subtracting the mean baseline memory score from the estimated mean memory score at each study wave. Estimates were obtained adjusting for baseline covariates (age at baseline, sex/gender, race and ethnicity, educational attainment, birth in southern region, and personality traits), time-varying covariates (household wealth per person, employment status, social contact [marital status, participation in social activities, and contact with children, other family members, or friends], smoking status, frequency of vigorous physical activity, self-reported diagnosed comorbidities [diabetes, hypertension, and stroke], self-rated health, self-reported pain, depressive symptoms measured by CES-D score, and activities of daily living limitations), and memory function in previous waves. Estimates for the sustained loneliness intervention are not shown for 2 years since the baseline because they correspond to the estimates for the baseline loneliness intervention. Some values for percent reduction rounded to 0.00 while still producing a small non-zero percent reduction. Sampling weights for community-dwelling adults in 2006 were used.

In analyses stratified by baseline age, sex/gender, and social isolation at baseline, we did not find evidence of an association between either the baseline or sustained loneliness intervention and memory function in any subgroup (Tables S7-S9).

The direction and magnitude of the estimated effects were consistent with those from the main analyses in sensitivity analyses using the 3-item scale of the Revised UCLA Loneliness Scale (Table S10), applying inverse probability of censoring weighting (Table S11), without using sampling weights (Table S12), including only one participant within a household (Table S13), and using 10 imputed datasets (Table S14). We also found that the observed mean memory score change and the estimated memory score change were almost identical over follow-up (Table S15).

## Discussion

We estimated the impacts of baseline and sustained hypothetical loneliness interventions on memory scores at the population level by applying the target trial emulation framework and TMLE to a nationally representative cohort of US middle-aged and older adults. We did not find evidence that either the baseline or sustained loneliness intervention was associated with better memory function over 12 years compared to the natural course. Our findings indicate that baseline and sustained interventions to ameliorate loneliness may be inefficient in protecting late-life memory function at the population level.

This study builds on previous research on the association between persistent loneliness and cognitive function and dementia risk. A recent study using HRS showed that longer duration of loneliness was associated with lower memory scores and faster memory decline among middle-aged and older adults in the United States.^10^ Although this study accounted for time-varying confounding using marginal structural models as a sensitivity analysis and confirmed the association between persistent loneliness and memory function, some potentially important time-varying confounders, such as health-related behaviors and comorbidities, were left unadjusted for. A study conducted in China showed that people aged 65 and older with persistent loneliness had lower Modified Mini-Mental State Examination scores 6 years after baseline than those without loneliness.^59^ Another study using the data from the Framingham Heart Study found that compared to no loneliness, persistent loneliness (loneliness reported by a participant at two successive waves) was associated with a higher risk of dementia.^60^

To our knowledge, the present study is the first to estimate the effects of hypothetical interventions to eliminate loneliness on population-average memory function. Notably, our loneliness interventions compared a scenario where loneliness was eliminated in the population with the natural course. Given that a large proportion of the sample (62.6%) never experienced loneliness during the follow-up, our contrasts are less extreme than the typical analysis that compares outcomes under scenarios in which either all individuals experienced loneliness or no individuals experienced loneliness. Our analysis likely yields estimates that better map onto expected outcomes under real-world population-level loneliness interventions as compared to the more extreme scenarios that are more commonly estimated. Our approach also accounted for an extensive set of potential time-varying confounders, including potential reverse causation between loneliness and memory function. This difference in causal estimands, as well as our approach to controlling for time-varying confounding, may explain why we did not find evidence that either of the baseline and sustained loneliness interventions was associated with better memory function, despite previous research showing a link between loneliness and memory function and decline.^10-13^

It is important to note that we estimated the effects of “hypothetical” interventions to eliminate loneliness from the population (vs targeted interventions to those identified as experiencing loneliness) and did not present an actual intervention that could eliminate loneliness. Although some potential interventions are suggested to mitigate loneliness (eg, cognitive behavioral therapy),^14-16^ a specific intervention that eliminates loneliness is yet to be identified. Also, it is worth noting that loneliness is a subjective feeling of a lack of social connection, while social isolation is an objective measure of limited social contacts.^2^ Thus, interventions to mitigate social isolation, such as programs to facilitate social engagement, may not necessarily ameliorate loneliness, although they may do so indirectly by enhancing individuals’ actual social connections. Under the consistency assumption that eliminating loneliness through any available means would have the same effect on memory function, our findings can be of relevance to policymakers and public health practitioners because our study indicates that baseline and sustained interventions to ameliorate loneliness in the entire population may be inefficient in protecting late-life memory function.

Our study has limitations. First, as an observational study, there may be unmeasured confounding, although we accounted for a wide range of potential confounders, including time-varying confounders and reverse causation between loneliness and memory function. Also, we cannot rule out the possibility that our causal modeling was not correct, potentially inducing bias in our estimates. Second, the missing at random assumption for multiple imputation may not hold if, for example, socially isolated participants eligible for the Leave-Behind Questionnaire were more likely to decline to answer questions related to social isolation. Finally, we used a cohort that represented middle-aged and older adults in the United States; therefore, our findings may not be generalizable to other populations such as younger populations and people outside the United States.

In conclusion, we did not find evidence that hypothetical interventions to reduce loneliness—either at baseline or sustained over time—would protect late-life memory function at the population level. Although we did not identify subpopulations that might benefit from the interventions based on baseline age, sex/gender, or social isolation status, further research to clarify whether other population subgroups might experience benefits could help inform more targeted intervention strategies.

## Supplementary Material

Web_Material_kwag044

## Data Availability

HRS data are publicly available at https://hrs.isr.umich.edu/dataproducts. Dataset construction and analysis code is available on Github: https://github.com/Mayeda-Research-Group/hypothetical_loneliness_intervention.

## References

[1] Prohaska T, Burholt V, Burns A, et al. Consensus statement: loneliness in older adults, the 21st century social determinant of health? *BMJ Open*. 2020;10(8):e034967. 10.1136/bmjopen-2019-034967 PMC7422633 32788184

[2] National Academies of Sciences Engineering, Medicine . Social Isolation and Loneliness in Older Adults: Opportunities for the Health Care System. National Academies Press; 2020. 32510896

[3] Heinrich LM, Gullone E. The clinical significance of loneliness: a literature review. *Clin Psychol Rev*. 2006;26(6):695-718. 10.1016/j.cpr.2006.04.002 16952717

[4] Cigna Corperation . The Loneliness Epidemic Persists: A Post-Pandemic Look at the State of Loneliness among U.S. Adults. 2021.

[5] Crowe CL, Domingue BW, Graf GH, et al. Associations of loneliness and social isolation with health span and life span in the U.S. Health and Retirement Study. *J Gerontol A Biol Sci Med Sci*. 2021;76(11):1997-2006. 10.1093/gerona/glab128 33963758 PMC8514074

[6] Perissinotto CM, Stijacic Cenzer I, Covinsky KE. Loneliness in older persons: a predictor of functional decline and death. *Arch Intern Med*. 2012;172(14):1078-1083. 10.1001/archinternmed.2012.1993 22710744 PMC4383762

[7] Ong AD, Uchino BN, Wethington E. Loneliness and health in older adults: a mini-review and synthesis. *Gerontology.* 2016;62(4):443-449. 10.1159/000441651 26539997 PMC6162046

[8] Theeke LA . Predictors of loneliness in U.S. adults over age sixty-five. *Arch Psychiatr Nurs*. 2009;23(5):387-396. 10.1016/j.apnu.2008 19766930

[9] Wilson C, Moulton B. Loneliness Among Older Adults: A National Survey of Adults 45+. AARP; 2010.

[10] Yu X, Westrick AC, Kobayashi LC. Cumulative loneliness and subsequent memory function and rate of decline among adults aged ≥50 in the United States, 1996 to 2016. *Alzheimers Dement*. 2023;19(2):578-588. 10.1002/alz.064679 35920364 PMC9895124

[11] Donovan NJ, Wu Q, Rentz DM, et al. Loneliness, depression and cognitive function in older U.S. adults. *Int J Geriatr Psychiatry*. 2017;32(5):564-573. 10.1002/gps.4495 27162047 PMC5102822

[12] Yin J, Lassale C, Steptoe A, et al. Exploring the bidirectional associations between loneliness and cognitive functioning over 10 years: the English longitudinal study of ageing. *Int J Epidemiol*. 2019;48(6):1937-1948. 10.1093/ije/dyz085 31056641 PMC6929532

[13] Sutin AR, Stephan Y, Luchetti M, et al. Loneliness and risk of dementia. *J Gerontol B Psychol Sci Soc Sci*. 2020;75(7):1414-1422. 10.1093/geronb/gby112 30365023 PMC7424267

[14] Masi CM, Chen HY, Hawkley LC, et al. A meta-analysis of interventions to reduce loneliness. *Pers Soc Psychol Rev*. 2011;15(3):219-266. 10.1177/1088868310377394 20716644 PMC3865701

[15] Mann F, Bone JK, Lloyd-Evans B, et al. A life less lonely: the state of the art in interventions to reduce loneliness in people with mental health problems. *Soc Psychiatry Psychiatr Epidemiol*. 2017;52(6):627-638. 10.1007/s00127-017-1392-y 28528389 PMC5487590

[16] Shekelle PG, Miake-Lye IM, Begashaw MM, et al. Interventions to reduce loneliness in community-living older adults: a systematic review and meta-analysis. *J Gen Intern Med*. 2024;39(6):1015-1028. 10.1007/s11606-023-08517-5 38200279 PMC11074098

[17] Rajan KB, Weuve J, Barnes LL, et al. Population estimate of people with clinical Alzheimer's disease and mild cognitive impairment in the United States (2020-2060). *Alzheimers Dement*. 2021;17(12):1966-1975. 10.1002/alz.12362 34043283 PMC9013315

[18] Iso-Markku P, Aaltonen S, Kujala UM, et al. Physical activity and cognitive decline among older adults: a systematic review and meta-analysis. *JAMA Netw Open*. 2024;7(2):e2354285. 10.1001/jamanetworkopen.2023.54285 38300618 PMC10835510

[19] Reitz C, Luchsinger J, Tang MX, et al. Effect of smoking and time on cognitive function in the elderly without dementia. *Neurology.* 2005;65(6):870-875. 10.1212/01.wnl.0000176057.22827.b7 16186526 PMC2669791

[20] Zeki Al Hazzouri A, Caunca MR, Nobrega JC, et al. Greater depressive symptoms, cognition, and markers of brain aging: northern Manhattan study. *Neurology.* 2018;90(23):e2077-e2085. 10.1212/WNL.0000000000005639 29743209 PMC5993180

[21] Sabia S, Marmot M, Dufouil C, et al. Smoking history and cognitive function in middle age from the Whitehall II study. *Arch Intern Med*. 2008;168(11):1165-1173. 10.1001/archinte.168.11.1165 18541824 PMC2696613

[22] Hawkley LC, Thisted RA, Cacioppo JT. Loneliness predicts reduced physical activity: cross-sectional & longitudinal analyses. *Health Psychol*. 2009;28(3):354-363. 10.1037/a0014400 19450042 PMC2791498

[23] Dyal SR, Valente TW. A systematic review of loneliness and smoking: small effects, big implications. *Subst Use Misuse*. 2015;50(13):1697-1716. 10.3109/10826084.2015.1027933 26555089 PMC4803029

[24] Okely JA, Deary IJ. Longitudinal associations between loneliness and cognitive ability in the Lothian birth cohort 1936. *J Gerontol B Psychol Sci Soc Sci*. 2019;74(8):1376-1386. 10.1093/geronb/gby086 30053217 PMC6777773

[25] Zhong BL, Chen SL, Tu X, et al. Loneliness and cognitive function in older adults: findings from the Chinese longitudinal healthy longevity survey. *J Gerontol B Psychol Sci Soc Sci*. 2017;72(1):120-128. 10.1093/geronb/gbw037 27013536 PMC5156491

[26] Mansournia MA, Etminan M, Danaei G, et al. Handling time varying confounding in observational research. *BMJ.* 2017;359:j4587. 10.1136/bmj.j4587 29038130

[27] Hernan MA, Robins JM. G-methods for time-varying treatments. In: Causal Inference: What If. Boca Raton, FL: CRC. 2020.

[28] Robins JM, Hernan MA, Brumback B. Marginal structural models and causal inference in epidemiology. *Epidemiology.* 2000;11(5):550-560. 10.1097/00001648-200009000-00011 10955408

[29] Naimi AI, Cole SR, Kennedy EH. An introduction to g methods. *Int J Epidemiol*. 2017;46(2):756-762. 10.1093/ije/dyw323 28039382 PMC6074945

[30] Rudolph JE, Cartus A, Bodnar LM, et al. The role of the natural course in causal analysis. *Am J Epidemiol*. 2022;191(2):341-348. 10.1093/aje/kwab248 34643230 PMC8897990

[31] Hernán MA, Robins JM. Using big data to emulate a target trial when a randomized trial is not available. *Am J Epidemiol*. 2016;183(8):758-764. 10.1093/aje/kwv254 26994063 PMC4832051

[32] Rojas-Saunero LP, Labrecque JA, Swanson SA. Invited commentary: conducting and emulating trials to study effects of social interventions. *Am J Epidemiol*. 2022;191(8):1453-1456. 10.1093/aje/kwac066 35445692 PMC9347019

[33] Sonnega A, Faul JD, Ofstedal MB, et al. Cohort profile: the health and retirement study (HRS). *Int J Epidemiol*. 2014;43(2):576-585. 10.1093/ije/dyu067 24671021 PMC3997380

[34] Smith J, Ryan LH, Fisher GG, et al. HRS Psychosocial and Lifestyle Questionnaire 2006-2016. Survey Research Center, Institute for Social Research, University of Michigan; 2017:202006-202016.

[35] Kotwal AA, Cenzer IS, Waite LJ, et al. A single question assessment of loneliness in older adults during the COVID-19 pandemic: a nationally-representative study. *J Am Geriatr Soc*. 2022;70(5):1342-1345. 10.1111/jgs.17700 35141875 PMC9106870

[36] Hughes ME, Waite LJ, Hawkley LC, et al. A short scale for measuring loneliness in large surveys: results from two population-based studies. *Res Aging*. 2004;26(6):655-672. 10.1177/0164027504268574 18504506 PMC2394670

[37] Wu Q, Tchetgen Tchetgen EJ, Osypuk TL, et al. Combining direct and proxy assessments to reduce attrition bias in a longitudinal study. *Alzheimer Dis Assoc Disord*. 2013;27(3):207-212. 10.1097/WAD.0b013e31826cfe90 22992720 PMC3731387

[38] Jorm AF . A short form of the informant questionnaire on cognitive decline in the elderly (IQCODE): development and cross-validation. *Psychol Med*. 1994;24(1):145-153. 10.1017/s003329170002691x 8208879

[39] Jorm AF, Christensen H, Korten AE, et al. Informant ratings of cognitive decline in old age: validation against change on cognitive tests over 7 to 8 years. *Psychol Med*. 2000;30(4):981-985. 10.1017/s0033291799002299 11037106

[40] Sutin AR, Stephan Y, Luchetti M, et al. Five-factor model personality traits and cognitive function in five domains in older adulthood. *BMC Geriatr*. 2019;19(1):343. 10.1186/s12877-019-1362-1 31805866 PMC6896269

[41] Lachman ME, Weaver SL. The Midlife Development Inventory (MIDI) Personality Scales: Scale Construction and Scoring. Vol. Vol. 7. Brandeis University; 1997:1-9.

[42] Steptoe A, Shankar A, Demakakos P, et al. Social isolation, loneliness, and all-cause mortality in older men and women. *Proc Natl Acad Sci U S A*. 2013;110(15):5797-5801. 10.1073/pnas.1219686110 23530191 PMC3625264

[43] Loeffler A, Steptoe A. Bidirectional longitudinal associations between loneliness and pain, and the role of inflammation. *Pain.* 2021;162(3):930-937. 10.1097/j.pain.0000000000002082 32960533 PMC7886943

[44] Kotwal AA, Cenzer IS, Waite LJ, et al. The epidemiology of social isolation and loneliness among older adults during the last years of life. *J Am Geriatr Soc*. 2021;69(11):3081-3091. 10.1111/jgs.17366 34247388 PMC8595510

[45] Whitlock EL, Diaz-Ramirez LG, Glymour MM, et al. Association between persistent pain and memory decline and dementia in a longitudinal cohort of elders. *JAMA Intern Med*. 2017;177(8):1146-1153. 10.1001/jamainternmed.2017.1622 28586818 PMC5588896

[46] Radloff LS . The CES-D scale: a self-report depression scale for research in the general population. *Appl Psychol Measur*. 1977;1(3):385-401. 10.1177/014662167700100306

[47] Smith BJ, Lim MH, Manera KE, et al. Bidirectional relationships between loneliness, social isolation, and physical inactivity in the household, income and labour dynamics in Australia cohort study. *Ann Behav Med*. 2024;58(9):619-627. 10.1093/abm/kaae043 39066664 PMC11305128

[48] Wootton RE, Greenstone HSR, Abdellaoui A, et al. Bidirectional effects between loneliness, smoking and alcohol use: evidence from a Mendelian randomization study. *Addiction.* 2021;116(2):400-406. 10.1111/add.15142 32542815

[49] Livingston G, Huntley J, Sommerlad A, et al. Dementia prevention, intervention, and care: 2020 report of the lancet commission. *Lancet.* 2020;396(10248):413-446. 10.1016/S0140-6736(20)30367-6 32738937 PMC7392084

[50] Schuler MS, Rose S. Targeted maximum likelihood estimation for causal inference in observational studies. *Am J Epidemiol*. 2017;185(1):65-73. 10.1093/aje/kww165 27941068

[51] Schomaker M, Luque-Fernandez MA, Leroy V, et al. Using longitudinal targeted maximum likelihood estimation in complex settings with dynamic interventions. *Stat Med*. 2019;38(24):4888-4911. 10.1002/sim.8340 31436859 PMC6800798

[52] Van der Laan MJ, Rose S. Targeted Learning. Springer; 2011.

[53] Williams N, Díaz I. lmtp: an R package for estimating the causal effects of modified treatment policies. *Observational Studies*. 2023;9(2):103-122. 10.1353/obs.2025.a973072 PMC13155344 42110222

[54] Naimi AI, Balzer LB. Stacked generalization: an introduction to super learning. *Eur J Epidemiol*. 2018;33(5):459-464. 10.1007/s10654-018-0390-z 29637384 PMC6089257

[55] van der Laan MJ, Polley EC, Hubbard AE. Super learner. *Stat Appl Genet Mol Biol*. 2007;6(1):Article25. 10.2202/1544-6115.1309 17910531

[56] Shaw C, Wu Y, Zimmerman SC, et al. Comparison of imputation strategies for incomplete longitudinal data in lifecourse epidemiology. *Am J Epidemiol*. 2023;192(12):2075-2084. 10.1093/aje/kwad139 37338987 PMC10988225

[57] Van Buuren S . Flexible Imputation of Missing Data. CRC press; 2018.

[58] van Buuren S, Groothuis-Oudshoorn K. Mice: multivariate imputation by chained equations in R. *J Stat Softw*. 2011;45(3):1-67. 10.18637/jss.v045.i03

[59] Zhong BL, Chen SL, Conwell Y. Effects of transient versus chronic loneliness on cognitive function in older adults: findings from the Chinese longitudinal healthy longevity survey. *Am J Geriatr Psychiatry*. 2016;24(5):389-398. 10.1016/j.jagp.2015.12.009 26905049 PMC4846538

[60] Akhter-Khan SC, Tao Q, Ang TFA, et al. Associations of loneliness with risk of Alzheimer's disease dementia in the Framingham heart study. *Alzheimers Dement*. 2021;17(10):1619-1627. 10.1002/alz.12327 33760348 PMC8460688

