# Supplementary material for: Estimating the effects of hypothetical loneliness interventions on memory function among middle-aged and older adults in the United States
Source: Am J Epidemiol. 2026 Mar 9;195(7):1823–33. doi: 10.1093/aje/kwag044 (PMC13343386; doi:10.1093/aje/kwag044)
Supplement: Web_Material_kwag044 [file web_material_kwag044.zip › MS_supp Hypothetical loneliness interventions and memory function_R3_clean.docx]

**Online Supplement**

Estimating the effects of hypothetical loneliness interventions on memory function among middle-aged and older adults in the United States

Ryo Ikesu, Yingyan Wu, L. Paloma Rojas-Saunero, Roch A. Nianogo, Jacqueline M. Torres, Ashwin Kotwal, Christina M. Ramirez, Yusuke Tsugawa, Elizabeth Rose Mayeda

Table of Contents

[Supplementary Methods 3](#_Toc222303132)

[Figure S1. Flowchart of the sample selection 7](#_Toc222303133)

[Table S1. List of auxiliary variables for multiple imputation 8](#_Toc222303134)

[Table S2. Percentage of missing values in auxiliary variables used in multiple imputations 9](#_Toc222303135)

[Table S3. Percentage of missing values for loneliness and memory scores in the main analytic sample in 2006–2018 (n=10,136) 10](#_Toc222303136)

[Table S4. Baseline characteristics of the sample, stratified by baseline loneliness status (sampling weights were applied) 11](#_Toc222303137)

[Table S5. Baseline characteristics of the sample after imputation, stratified by baseline loneliness status 15](#_Toc222303138)

[Table S6. Pattern of loneliness in the sample during follow-up, stratified by baseline loneliness status 17](#_Toc222303139)

[Table S7. Estimated mean change in memory scores from baseline under natural course and hypothetical loneliness interventions, stratified by baseline age 18](#_Toc222303140)

[Table S8. Estimated mean change in memory scores from baseline under natural course and hypothetical loneliness interventions, stratified by sex/gender 20](#_Toc222303141)

[Table S9. Estimated mean change in memory scores from baseline under natural course and hypothetical loneliness interventions, stratified by social isolation status at baseline 22](#_Toc222303142)

[Table S10. Estimated mean change in memory scores from baseline under natural course and hypothetical loneliness interventions, using the 3-item UCLA loneliness scale 24](#_Toc222303143)

[Table S11. Estimated mean change in memory scores from baseline under natural course and hypothetical loneliness interventions, estimated with inverse probability of censoring weighting for community-dwelling HRS participants at 2006 (N = 17,105) 25](#_Toc222303144)

[Table S12. Estimated mean change in memory scores from baseline under natural course and hypothetical loneliness interventions, without applying sampling weights 26](#_Toc222303145)

[Table S13. Estimated mean change in memory scores from baseline under natural course and hypothetical loneliness interventions, after limiting the sample to only one participant within a household (N = 7,627) 27](#_Toc222303146)

[Table S14. Estimated mean change in memory scores from baseline under natural course and hypothetical loneliness interventions, estimated with ten imputed datasets 28](#_Toc222303147)

[Table S15. Observed mean memory change over follow-up and mean memory change over follow-up estimated from TMLE under the natural course 29](#_Toc222303148)

# **Supplementary Methods**

*Adjustment for Birth in the Southern US Region and Personality Trait*

We adjusted for birth in the southern US region because of its link to cardiovascular conditions, which may lead to loneliness, and dementia incidence (1-3). We adjusted for personality traits because they are potentially associated with both loneliness and memory function (4, 5). Personality traits were extracted from the leave-behind questionnaire, defined using the 2006 questionnaire for approximately half the sample and the 2008 questionnaire for the other half, assuming personality traits do not change meaningfully over a short time period.

*Adjustment for Memory Scores*

In this study, memory scores were treated not only as the outcome variable but also as a time-varying confounder, given the potential bidirectional relationship between loneliness and memory function (6-8). We adjusted for concurrent memory function as a confounder (vs. a mediator) because we expect that there should be some elapsed time for loneliness to affect memory function. For example, loneliness may cause depressive symptoms through negative cognitive patterns (e.g., diminished self-confidence) and increased stress levels (9). In addition, to cope with stress induced by loneliness, people who feel lonely may engage in health-damaging behaviors, such as physical inactivity and smoking (10, 11). These consequences of loneliness may negatively impact memory function (12-15), arguably with some elapsed time between loneliness and memory function.

*Causal Estimands*

To assess the effectiveness of the baseline and sustained loneliness interventions, we compared counterfactual memory scores at each study wave over 12 years after baseline under the baseline and sustained interventions to the natural course by fitting separate targeted maximum likelihood estimation (TMLE) models for each wave during the follow-up. The estimate for the natural course was obtained as the observed mean of the memory score after multiple imputation. The causal estimands under these scenarios were:
Population average memory score at wave t under the sustained loneliness intervention:

$$\hat{E}[{Memor score}_{t}^{\bar{Loneliness}=0}]$$

Population average memory score at wave t under the baseline loneliness intervention:

$$\hat{E}[{Memor score}_{t}^{Baseline loneliness=0}]$$

Population average memory score at wave t under the natural course:

$$E[{Memor score}_{t}^{\bar{Loneliness}=As observed}]$$

where $\bar{Loneliness}$ denotes loneliness history and $\hat{E}[\boldsymbol{\cdot}]$ denotes the population average memory score estimated from TMLE for the baseline and sustained loneliness interventions.

*Assumptions of Conditional Exchangeability, Consistency, and Positivity*

TMLE provides valid effect estimates under several assumptions, including conditional exchangeability (or no unmeasured confounding), consistency, and positivity. For conditional exchangeability, we assumed all common causes of both the exposure and the outcome were measured. In this study, the consistency assumption means that eliminating loneliness through any available means would have the same effect on memory function (16). The positivity assumption requires that the probability of exposure/treatment is non-zero for every combination of covariates, and there are two types of violations of the assumption: (1) structural violations and (2) random violations (17). Our assessment of the positivity assumption was based on baseline covariate distributions, subject-matter knowledge about the plausibility of structural violations, and the use of TMLE to address random sparsity in covariate strata. In structural violations of positivity, individuals with certain sets of covariate values cannot be treated (or not treated). For example, if there is a contraindication for a certain medical treatment (e.g., conducting a high-risk open heart surgery can be contraindicated for people with limited activities of daily living), the probability of receiving treatment (e.g., high-risk open heart surgery) should be zero–i.e., positivity violations– for people with the specific condition (e.g., limited activities of daily living). When there is a structural violation due to a certain covariate, certain strata defined by that covariate will have zero observations. From the table of sample characteristics (**Table 2**), there was no cell indicating structural violations of positivity. More importantly, we believe there is no plausible mechanism that causes a structural violation of the positivity assumption for our exposure (loneliness).

On the other hand, random violations of positivity can occur in a finite sample even without a structural violation of positivity. That is, if the analytic sample is stratified on several covariates, it is possible to have combinations of covariate values with zero observations, even if the probability of treatment is not truly zero in the target population. In the presence of random violations, a modeling approach, such as the TMLE used in this study, can be employed to estimate the probability of exposure in strata with random zeroes using data from individuals in other strata. Overall, we believe that violations of the positivity assumption are not concerning in our study.

*Missing Data Handling*

We applied multilevel multiple imputation (5 imputed datasets) to handle missing data in longitudinal data, using the fully conditional specification approach using the R package mice (18-20). To obtain unbiased estimates with multiple imputation, the probability of missingness needs to be at random, conditional on observed variables (i.e., the missing at random assumption). Multiple imputation was conducted with 20 Markov chain Monte Carlo (MCMC) iterations for each imputed dataset, and we visually confirmed that the MCMC iterations achieved model convergence. The predictive mean matching (PMM) method was used for both continuous and categorical variables with missingness, because PMM is shown to be robust to bias due to missing not at random in longitudinal data (18). Beyond the variables listed above, auxiliary variables were included in imputation models to help fulfill the missing at random assumption for multiple imputation (details in **Table S1**). In addition, to boost the performance of the imputation of missing values in the longitudinal data, we included Health and Retirement Study (HRS) 2020 data in the multiple imputation as it may be predictive of missing variables in the previous waves (HRS 2020 data were not used in the analyses). We combined estimates using Rubin’s rules (21).

# **Figure S1. Flowchart of the sample selection**

Health Retirement Study participants who were eligible for the core interview in 2006 (n = 18,469)

Community-dwelling participants with non-zero sampling weights aged 50 and older in 2006 (n = 17,105)

Participants who were still alive in 2018 (n = 10,136)

# **Table S1. List of auxiliary variables for multiple imputation**

| **Category** | **Variables** | **Justification for exclusion from TMLE** |
| --- | --- | --- |
| Demographic characteristic | - Household income per person (household income in US dollars divided by square root of the number of household members - Interview by proxy respondent (yes vs. no) | - Household wealth per person was adjusted instead, as it better captures long-term financial status in older adults. Including both may introduce multicollinearity and redundancy, with limited added value for confounding control. - The composite memory score (outcome of interest) was estimated from a model that includes an indicator for whether the interview was conducted by a proxy respondent. Additionally, social contact, which is a better measure of social relationship than having a proxy respondent, was adjusted for. |
| Health conditions | - Self-reported diagnosed comorbidities (cancer, lung disease, heart disease, psychiatric problems, arthritis; yes vs. no) - Self-reported body mass index - Dementia probability score^a^ | - The impacts of these additional health conditions were likely captured through the health conditions adjusted for (self-rated health, disability status, pain, depressive symptoms, diabetes, hypertension, stroke). - The impacts of body mass index were likely captured through the health conditions adjusted for and physical activity. - We conceptualize dementia probability scores to reflect deterioration of the outcome (memory function). |
| Use of healthcare services | - Hospital stay in previous 2 years (yes vs. no) - Lived in a nursing home at interview (yes vs. no) - Doctor visit in previous 2 years (yes vs. no) - Medical expenditures in the previous 2 years (out of pocket) (US dollars) | - These variables were likely proxy variables for health conditions. Important health conditions for the loneliness-memory function relationship were adjusted for. |

These variables were treated as time-varying variables and used only for multiple imputations.

^a^Dementia probability score using the Wu algorithm to predict dementia status (22); the model was developed in the Aging, Demographics, and Memory Study (ADAMS), a sub-study of HRS, and can be applied to estimate dementia probability scores for all HRS participants
Abbreviation: TMLE = targeted maximum likelihood estimation.

# **Table S2. Percentage of missing values in auxiliary variables used in multiple imputations**

| **Characteristics** | **Percent missing (%)**^a^ |
| --- | --- |
| Household income per person | 9.63 |
| Cancer (self-reported) | 9.63 |
| Lung disease (self-reported) | 9.63 |
| Heart disease (self-reported) | 9.63 |
| Psychiatric problems (self-reported) | 9.63 |
| Arthritis (self-reported) | 9.63 |
| Lived in nursing home at interview | 9.63 |
| Medical expenditures in previous 2 years (out of pocket) | 9.63 |
| Proxy informant | 9.63 |
| Doctor visit in previous 2 years | 9.92 |
| Hospital stay in previous 2 years | 10.06 |
| Self-reported body mass index | 10.77 |
| Dementia probability | 32.85 |

^a^Numbers of percent missing were calculated for the longitudinal data used for the multiple imputation. To boost the performance of the imputation of missing values in the longitudinal data, we included the HRS 2020 data in the multiple imputation as it may be predictive of missing variables in the previous waves (the HRS 2020 data were not used in the analyses).

Abbreviation: HRS = Health and Retirement Study.

# **Table S3. Percentage of missing values for loneliness and memory scores in the main analytic sample in 2006–2018 (n=10,136)**

| **Year (wave)** | **Loneliness (%)** | **Memory score (%)** |
| --- | --- | --- |
| 2006 (wave 1) | 3.8 | 12.2 |
| 2008 (wave 2) | 6.4 | 14.3 |
| 2010 (wave 3) | 8.4 | 15.4 |
| 2012 (wave 4) | 9.5 | 16.4 |
| 2014 (wave 5) | 12.5 | 18.4 |
| 2016 (wave 6) | 18.2 | 22.8 |
| 2018 (wave 7) | NA | 38.3 |

# **Table S4. Baseline characteristics of the sample, stratified by baseline loneliness status (sampling weights were applied)**

|  | **Total** | **Baseline loneliness** | | |
| --- | --- | --- | --- | --- |
|  |  | **No** | **Yes** | **Missing** |
| Unweighted N | 10,136 | 8,293 | 1,461 | 382 |
| Weighted N ^a^ | 50,209,090 | 41,277,864 | 7,024,598 | 1,906,628 |
| Age (median [Q1, Q3]) | 60 [56, 67] | 60 [56, 67] | 60 [56, 67] | 60 [56, 65] |
| Missing | 0.00% | 0.00% | 0.00% | 0.00% |
| Women (%) | 27,969,761 (55.7) | 22,929,861 (55.6) | 4,621,328 (65.8) | 418,572 (22.0) |
| Missing | 0.00% | 0.00% | 0.00% | 0.00% |
| Race and ethnicity (%) |  |  |  |  |
| Non-Hispanic White | 39,921,833 (79.5) | 33,933,678 (82.2) | 4,657,916 (66.3) | 1,330,239 (69.8) |
| Non-Hispanic Black | 4,478,012 (8.9) | 3,327,175 (8.1) | 976,270 (13.9) | 174,567 (9.2) |
| Hispanic | 4,242,276 (8.4) | 2,856,719 (6.9) | 1,111,923 (15.8) | 273,634 (14.4) |
| Other | 1,566,969 (3.1) | 1,160,292 (2.8) | 278,489 (4.0) | 128,188 (6.7) |
| Missing | 0.00% | 0.00% | 0.00% | 0.00% |
| Educational attainment (%) |  |  |  |  |
| Less than high school degree | 6,770,058 (13.5) | 4,616,560 (11.2) | 1,682,595 (24.0) | 470,903 (24.7) |
| GED | 2,148,747 (4.3) | 1,684,969 (4.1) | 404,251 (5.8) | 59,527 (3.1) |
| High school graduate | 14,580,640 (29.0) | 12,084,686 (29.3) | 1,968,371 (28.0) | 527,583 (27.7) |
| Some college | 12,595,407 (25.1) | 10,450,690 (25.3) | 1,768,953 (25.2) | 375,764 (19.7) |
| College graduate | 14,108,455 (28.1) | 12,435,176 (30.1) | 1,200,428 (17.1) | 472,851 (24.8) |
| Missing | 0.01% | 0.01% | 0.00% | 0.00% |
| Birth in southern US region (%) ^b^ | 14,355,967 (28.6) | 11,449,479 (27.8) | 2,243,263 (31.9) | 663,225 (34.8) |
| Missing | 0.05% | 0.05% | 0.05% | 0.14% |
| Measures of personality ^c^ |  |  |  |  |
| Neuroticism (median [Q1, Q3]) | 3 [2, 3] | 3 [2, 4] | 2 [2, 3] | 3 [2, 3] |
| Missing | 16.58% | 14.19% | 19.56% | 57.38% |
| Extraversion (median [Q1, Q3]) | 2 [1, 2] | 2 [1, 2] | 2 [1, 2] | 2 [1, 2] |
| Missing | 16.60% | 14.17% | 19.77% | 57.47% |
| Openness to experience  (median [Q1, Q3]) | 2 [2, 2] | 2 [2, 2] | 2 [2, 3] | 2 [2, 3] |
| Missing | 16.86% | 14.41% | 20.16% | 57.60% |
| Agreeableness (median [Q1, Q3]) | 1 [1, 2] | 1 [1, 2] | 1 [1, 2] | 2 [1, 2] |
| Missing | 16.59% | 14.17% | 19.75% | 57.47% |
| Conscientiousness (median [Q1, Q3]) | 2 [1, 2] | 2 [1, 2] | 2 [1, 2] | 2 [1, 2] |
| Missing | 16.64% | 14.21% | 19.80% | 57.54% |
| Household wealth per person  (median [Q1, Q3]) | 175,368 [50,205, 453,498] | 194,887 [61,165, 494,222] | 83,065 [8,715, 259,881] | 168,510 [55,228, 387,844] |
| Missing | 0.00% | 0.00% | 0.00% | 0.00% |
| Currently working (%) | 28,148,130 (56.1) | 24,032,481 (58.2) | 2,875,684 (40.9) | 1,239,965 (65.4) |
| Missing | 0.06% | 0.05% | 0.00% | 0.59% |
| Measures of social contact |  |  |  |  |
| Married or partnered (%) | 36,125,038 (72.0) | 31,177,092 (75.5) | 3,216,326 (45.8) | 1,731,620 (90.8) |
| Missing | 0.02% | 0.01% | 0.03% | 0.00% |
| Participation in social activities (monthly) (%) | 29,798,089 (80.7) | 25,065,220 (81.0) | 3,875,826 (77.4) | 857,043 (89.5) |
| Missing | 26.45% | 24.99% | 28.70% | 49.77% |
| Contact with children (monthly) (%) | 18,494,097 (85.4) | 15,961,388 (85.7) | 2,322,958 (83.9) | 209,751 (84.8) |
| Missing | 56.89% | 54.87% | 60.59% | 87.03% |
| Contact with other family members (monthly) (%) | 17,209,211 (77.9) | 14,831,127 (78.0) | 2,196,258 (78.3) | 181,826 (71.8) |
| Missing | 56.02% | 53.91% | 60.06% | 86.72% |
| Contact with friends (monthly) (%) | 18,765,477 (84.5) | 16,409,553 (85.8) | 2,174,242 (76.7) | 181,682 (75.0) |
| Missing | 55.78% | 53.67% | 59.63% | 87.30% |
| Socially isolated (%) ^d^ | 6,343,542 (30.2) | 5,098,593 (28.1) | 1,165,540 (44.7) | 79,409 (33.6) |
| Missing | 58.21% | 56.05% | 62.92% | 87.61% |
| Current smoking (%) | 6,902,307 (13.8) | 5,204,673 (12.7) | 1,415,173 (20.2) | 282,461 (15.1) |
| Missing | 0.40% | 0.38% | 0.21% | 1.66% |
| Vigorous physical activity at least once per month (%) | 23,824,537 (47.5) | 20,492,709 (49.7) | 2,426,143 (34.6) | 905,685 (47.6) |
| Missing | 0.11% | 0.09% | 0.16% | 0.20% |
| Self-reported diagnosed comorbidities (%) |  |  |  |  |
| Diabetes | 6,905,934 (13.8) | 5,219,206 (12.6) | 1,376,528 (19.6) | 310,200 (16.3) |
| Missing | 0.00% | 0.00% | 0.00% | 0.00% |
| Hypertension | 22,985,736 (45.8) | 18,356,932 (44.5) | 3,688,166 (52.5) | 940,638 (49.3) |
| Missing | 0.00% | 0.00% | 0.00% | 0.00% |
| Stroke | 1,775,980 (3.5) | 1,212,027 (2.9) | 452,765 (6.4) | 111,188 (5.8) |
| Missing | 0.00% | 0.00% | 0.00% | 0.00% |
| Self-rated health (%) |  |  |  |  |
| Excellent/very good | 25,835,257 (51.5) | 22,853,300 (55.4) | 2,098,304 (29.9) | 883,653 (46.3) |
| Good | 14,766,012 (29.4) | 12,194,975 (29.6) | 1,953,853 (27.9) | 617,184 (32.4) |
| Fair/poor | 9,574,437 (19.1) | 6,208,364 (15.0) | 2,960,282 (42.2) | 405,791 (21.3) |
| Missing | 0.07% | 0.05% | 0.17% | 0.00% |
| Moderate or severe pain (%) | 10,927,818 (21.8) | 7,756,017 (18.8) | 2,711,120 (38.7) | 460,681 (24.4) |
| Missing | 0.14% | 0.10% | 0.16% | 0.85% |
| CES-D score without loneliness item (median [Q1, Q3]) ^e^ | 0 [0, 2] | 0 [0, 1] | 3 [1, 5] | 4 [1, 6] |
| Missing | 4.23% | 0.37% | 1.27% | 98.71% |
| ADL score (median [Q1, Q3]) ^f^ | 0 [0, 0] | 0 [0, 0] | 0 [0, 1] | 0 [0, 0] |
| Missing | 0.04% | 0.02% | 0.00% | 0.78% |

Sampling weights for community-dwelling individuals in 2006 were applied. Percentages were calculated among non-missing values for each

characteristic.

^a^Weighted numbers of Health and Retirement Study participants were calculated with sampling weights for community-dwelling adults in 2006.

^b^Birth in southern US region was based on self-reported state of birth classified by US Census region, including Alabama, Arkansas, Delaware, Florida, Georgia, Kentucky, Louisiana, Maryland, Mississippi, North Carolina, Oklahoma, South Carolina, Tennessee, Texas, Virginia, Washington, D.C., and West Virginia.

^c^Measures of personality (neuroticism, extraversion, openness to experience, agreeableness, and conscientiousness) were scored based on 4-point scales ranging from 1 to 4 with higher scores more consistent with the corresponding trait.^d^Participants were considered socially isolated if they met at least two of the following five conditions: not married or partnered, less than monthly participation in social activities, and less than monthly contact with children, other family members, or friends.

^e^CES-D score without loneliness item; score range 0 to 7.

^f^ADL score was defined by assigning one point to each of any difficulty in dressing, eating, bathing and showering, walking across a room, and getting in and out of bed; score range 0 to 6.
Abbreviations: ADL = activities of daily living; CES-D = Center for Epidemiological Studies-Depression; GED = general educational development; HRS = Health and Retirement Study; Q1 = first quartile: Q3 = third quartile.

# **Table S5. Baseline characteristics of the sample after imputation, stratified by baseline loneliness status**

|  | **Total** | **Baseline loneliness** | |
| --- | --- | --- | --- |
|  |  | **No** | **Yes** |
| Unweighted N | 10,136 | 8,618 | 1,518 |
| Age (median [Q1, Q3]) | 64 [58, 70] | 65 [58, 70] | 64 [57, 70] |
| Women (%) | 6,140 (60.6) | 5,073 (58.9) | 1,067 (70.3) |
| Race and ethnicity (%) |  |  |  |
| Non-Hispanic White | 7,443 (73.4) | 6,553 (76.0) | 890 (58.6) |
| Non-Hispanic Black | 1,376 (13.6) | 1,082 (12.6) | 294 (19.4) |
| Hispanic | 1,048 (10.3) | 760 (8.8) | 288 (19.0) |
| Other | 269 (2.7) | 224 (2.6) | 45 (3.0) |
| Educational attainment (%) |  |  |  |
| Less than high school degree | 1,752 (17.3) | 1,305 (15.1) | 446 (29.4) |
| GED | 453 (4.5) | 368 (4.3) | 85 (5.6) |
| High school graduate | 3,119 (30.8) | 2,676 (31.0) | 443 (29.2) |
| Some college | 2,354 (23.2) | 2,023 (23.5) | 331 (21.8) |
| College graduate | 2,459 (24.3) | 2,246 (26.1) | 213 (14.0) |
| Birth in southern US region (%) ^b^ | 3,257 (32.1) | 2,718 (31.5) | 539 (35.5) |
| Measures of personality ^c^ |  |  |  |
| Neuroticism (median [Q1, Q3]) | 3 [2, 3] | 3 [3, 4] | 3 [2, 3] |
| Extraversion (median [Q1, Q3]) | 2 [1, 2] | 2 [1, 2] | 2 [1, 2] |
| Openness to experience  (median [Q1, Q3]) | 2 [2, 2] | 2 [2, 2] | 2 [2, 3] |
| Agreeableness (median [Q1, Q3]) | 1 [1, 2] | 1 [1, 2] | 1 [1, 2] |
| Conscientiousness (median [Q1, Q3]) | 2 [1, 2] | 2 [1, 2] | 2 [1, 2] |
| Household wealth per person  (median [Q1, Q3]) | 171,120 [47,343, 439,447] | 190,157 [60,000, 475,835] | 77,110 [5,669, 245,740] |
| Currently working (%) | 4,775 (47.1) | 4,261 (49.4) | 514 (33.9) |
| Measures of social contact |  |  |  |
| Married or partnered (%) | 7,191 (70.9) | 6,530 (75.8) | 660 (43.5) |
| Participation in social activities (monthly) (%) | 7,912 (78.1) | 6,759 (78.4) | 1,152 (75.9) |
| Contact with children (monthly) (%) | 8,777 (86.6) | 7,484 (86.8) | 1,293 (85.2) |
| Contact with other family members (monthly) (%) | 7,916 (78.1) | 6,752 (78.3) | 1,165 (76.7) |
| Contact with friends (monthly) (%) | 8,600 (84.9) | 7,402 (85.9) | 1,199 (79.0) |
| Socially isolated (%) ^d^ | 2,733 (27.0) | 2,127 (24.7) | 606 (39.9) |
| Current smoking (%) | 1,239 (12.2) | 983 (11.4) | 256 (16.8) |
| Vigorous physical activity at least once per month (%) | 4,548 (44.9) | 4,062 (47.1) | 486 (32.0) |
| Self-reported diagnosed comorbidities (%) |  |  |  |
| Diabetes | 1,563 (15.4) | 1,230 (14.3) | 333 (21.9) |
| Hypertension | 5,095 (50.3) | 4,235 (49.1) | 860 (56.7) |
| Stroke | 418 (4.1) | 318 (3.7) | 100 (6.6) |
| Self-rated health (%) |  |  |  |
| Excellent/very good | 4,948 (48.8) | 4,518 (52.4) | 430 (28.3) |
| Good | 3,130 (30.9) | 2,692 (31.2) | 438 (28.9) |
| Fair/poor | 2,058 (20.3) | 1,408 (16.3) | 650 (42.8) |
| Moderate or severe pain (%) | 2,221 (21.9) | 1,644 (19.1) | 577 (38.0) |
| CES-D score without loneliness item (median [Q1, Q3]) ^e^ | 0 [0, 2] | 0 [0, 1] | 3 [1, 5] |
| ADL score (median [Q1, Q3]) ^f^ | 0 [0, 0] | 0 [0, 0] | 0 [0, 1] |

This table averages the number of participants across imputed datasets.

^a^Birth in southern US region was based on self-reported state of birth classified by US Census region, including Alabama, Arkansas, Delaware, Florida, Georgia, Kentucky, Louisiana, Maryland, Mississippi, North Carolina, Oklahoma, South Carolina, Tennessee, Texas, Virginia, Washington, D.C., and West Virginia.

^b^Measures of personality (neuroticism, extraversion, openness to experience, agreeableness, and conscientiousness) were scored based on 4-point scales ranging from 1 to 4 with higher scores more consistent with the corresponding trait.

^c^Participants were considered socially isolated if they met at least two of the following five conditions: not married or partnered, less than monthly participation in social activities, and less than monthly contact with children, other family members, or friends.

^d^CES-D score without loneliness item; score range 0 to 7.

^e^ADL score was defined by assigning one point to each of any difficulty in dressing, eating, bathing and showering, walking across a room, and getting in and out of bed; score range 0 to 6.
Abbreviations: ADL = activities of daily living; CES-D = Center for Epidemiological Studies-Depression; GED = general educational development; HRS = Health and Retirement Study; Q1 = first quartile: Q3 = third quartile.

# **Table S6. Pattern of loneliness in the sample during follow-up, stratified by baseline loneliness status**

| **Loneliness pattern** | **Count (%)** |
| --- | --- |
| Total | 8,184 (100) |
| Persistent loneliness (Lonely at all waves) | 169 (2.1) |
| Persistent non-loneliness (Never lonely) | 5,122 (62.6) |
| Transient loneliness (Variability in loneliness across six waves) | 2,893 (35.3) |
| Baseline loneliness yes | 1,157 (100) |
| Persistent loneliness (Lonely at all waves) | 169 (14.6) |
| Transient loneliness (Variability in loneliness across six waves) | 988 (85.4) |
| Baseline loneliness no | 7,027 (100) |
| Persistent non-loneliness (Never lonely) | 5,122 (72.9) |
| Transient loneliness (Variability in loneliness across six waves) | 1,905 (27.1) |

This table is based on 8,184 Health and Retirement Study participants with loneliness assessed at all six waves (2006–2016).

# **Table S7. Estimated mean change in memory scores from baseline under natural course and hypothetical loneliness interventions, stratified by baseline age**

| **Years since baseline** | **Mean memory change from baseline** | | | | **Impact of interventions** | | | |
| --- | --- | --- | --- | --- | --- | --- | --- | --- |
|  |  |  |  |  | **Baseline loneliness intervention vs. natural course** | | **Sustained loneliness intervention vs. natural course** | |
|  | **Natural course**  **[A]**  **(95% CI)** | **Baseline loneliness intervention**  **[B]**  **(95% CI)** | **Sustained loneliness intervention**  **[C]**  **(95% CI)** | | **Difference**  **[B] - [A]**  **(95% CI)** | **Percentage reduction**  **100***  **([A] - [B])/[A]** | **Difference**  **[C] - [A]**  **(95% CI)** | **Percentage reduction**  **100***  **([A] - [C])/[A]** |
| **Age** $\boldsymbol{<}$ **65** | | | | | | | | |
| 2 years | -0.04  (-0.06 to -0.03) | -0.05  (-0.06 to -0.03) | | NA | 0.00  (-0.01 to 0.00) | -2.6 | NA | NA |
| 4 years | -0.12  (-0.14 to -0.11) | -0.12  (-0.14 to -0.11) | | -0.12  (-0.14 to -0.10) | 0.00  (-0.01 to 0.01) | -0.1 | 0.00  (-0.01 to 0.01) | 1.1 |
| 6 years | -0.19  (-0.21 to -0.18) | -0.19  (-0.21 to -0.18) | | -0.19  (-0.21 to -0.17) | 0.00  (-0.01 to 0.01) | -0.7 | 0.00  (-0.01 to 0.02) | 0.8 |
| 8 years | -0.24  (-0.26 to -0.22) | -0.24  (-0.26 to -0.23) | | -0.23  (-0.26 to -0.21) | 0.00  (-0.01 to 0.01) | -0.3 | 0.01  (-0.01 to 0.03) | 3.8 |
| 10 years | -0.35  (-0.37 to -0.33) | -0.35  (-0.37 to -0.32) | | -0.34  (-0.38 to -0.30) | 0.00  (-0.01 to 0.01) | 0.3 | 0.01  (-0.02 to 0.04) | 2.9 |
| 12 years | -0.41  (-0.43 to -0.39) | -0.41  (-0.43 to -0.39) | | -0.40  (-0.47 to -0.33) | 0.00  (-0.01 to 0.01) | -0.0 | 0.01  (-0.06 to 0.07) | 1.8 |
| **Age** $\boldsymbol{\geq}$ **65** | | | | | | | | |
| 2 years | -0.10  (-0.12 to -0.08) | -0.10  (-0.12 to -0.08) | | NA | 0.00  (-0.01 to 0.01) | -0.6 | NA | NA |
| 4 years | -0.29  (-0.31 to -0.26) | -0.29  (-0.32 to -0.26) | | -0.28  (-0.31 to -0.26) | 0.00  (-0.01 to 0.01) | -0.7 | 0.00  (-0.01 to 0.02) | 1.7 |
| 6 years | -0.43  (-0.45 to -0.40) | -0.42  (-0.45 to -0.40) | | -0.41  (-0.45 to -0.38) | 0.00  (-0.01 to 0.02) | 1.0 | 0.01  (-0.01 to 0.03) | 3.3 |
| 8 years | -0.59  (-0.62 to -0.56) | -0.59  (-0.62 to -0.56) | | -0.58  (-0.61 to -0.54) | 0.00  (-0.01 to 0.01) | -0.1 | 0.01  (-0.01 to 0.04) | 2.3 |
| 10 years | -0.83  (-0.86 to -0.80) | -0.83  (-0.87 to -0.80) | | -0.81  (-0.85 to -0.77) | 0.00  (-0.01 to 0.01) | -0.1 | 0.02  (0.00 to 0.05) | 2.7 |
| 12 years | -0.94  (-0.98 to -0.90) | -0.94  (-0.98 to -0.90) | | -0.90  (-0.96 to -0.85) | 0.00  (-0.02 to 0.01) | -0.2 | 0.04  (0.00 to 0.08) | 4.0 |

Estimated mean memory change from baseline was calculated by subtracting the mean baseline memory score from the estimated mean memory score at each study wave. Estimates were obtained adjusting for baseline covariates (age at baseline, sex/gender, race and ethnicity, educational attainment, whether born in southern states, and personality traits), time-varying covariates (household wealth per person, employment status, social contact [marital status, participation in social activities, and contact with children, other family members, or friends], smoking status, frequency of vigorous physical activity, self-reported diagnosed comorbidities [diabetes, hypertension, and stroke], self-rated health, self-reported pain, depressive symptoms measured by CES-D score, and activities of daily living limitations), and memory function in previous waves. Estimates for the sustained loneliness intervention are not shown for 2 years since the baseline because they correspond to the estimates for the baseline loneliness intervention. Some values for percent reduction rounded to 0.00 while still producing a small non-zero percent reduction. Sampling weights for community-dwelling adults in 2006 were used.

Abbreviations: CI = confidence interval; NA = not applicable.

# **Table S8. Estimated mean change in memory scores from baseline under natural course and hypothetical loneliness interventions, stratified by sex/gender**

| **Years since baseline** | **Mean memory change from baseline** | | | **Impact of interventions** | | | |
| --- | --- | --- | --- | --- | --- | --- | --- |
|  |  |  |  | **Baseline loneliness intervention vs. natural course** | | **Sustained loneliness intervention vs. natural course** | |
|  | **Natural course**  **[A]**  **(95% CI)** | **Baseline loneliness intervention**  **[B]**  **(95% CI)** | **Sustained loneliness intervention**  **[C]**  **(95% CI)** | **Difference**  **[B] - [A]**  **(95% CI)** | **Percentage reduction**  **100***  **([A] - [B])/[A]** | **Difference**  **[C] - [A]**  **(95% CI)** | **Percentage reduction**  **100***  **([A] - [C])/[A]** |
| **Women** | | | | | | | |
| 2 years | -0.07  (-0.08 to -0.05) | -0.07  (-0.09 to -0.05) | NA | 0.00  (-0.01 to 0.00) | -5.8 | NA | NA |
| 4 years | -0.18  (-0.20 to -0.16) | -0.18  (-0.20 to -0.16) | -0.18  (-0.20 to -0.16) | 0.00  (-0.01 to 0.01) | -0.3 | 0.01  (0.00 to 0.01) | 2.8 |
| 6 years | -0.27  (-0.29 to -0.25) | -0.27  (-0.30 to -0.25) | -0.27  (-0.29 to -0.24) | 0.00  (-0.01 to 0.01) | 0.4 | 0.01  (-0.01 to 0.02) | 2.3 |
| 8 years | -0.38  (-0.40 to -0.35) | -0.38  (-0.40 to -0.35) | -0.36  (-0.40 to -0.33) | 0.00  (-0.01 to 0.01) | -0.3 | 0.01  (-0.01 to 0.03) | 2.8 |
| 10 years | -0.53  (-0.56 to -0.50) | -0.54  (-0.57 to -0.51) | -0.52  (-0.56 to -0.47) | -0.01  (-0.02 to 0.01) | -1.0 | 0.01  (-0.02 to 0.05) | 2.6 |
| 12 years | -0.60  (-0.63 to -0.58) | -0.61  (-0.64 to -0.58) | -0.58  (-0.64 to -0.51) | -0.01  (-0.02 to 0.01) | -1.0 | 0.03  (-0.03 to 0.09) | 4.6 |
| **Men** | | | | | | | |
| 2 years | -0.06  (-0.08 to -0.03) | -0.06  (-0.08 to -0.03) | NA | 0.00  (-0.01 to 0.01) | -1.0 | NA | NA |
| 4 years | -0.17  (-0.19 to -0.14) | -0.17  (-0.20 to -0.14) | -0.17  (-0.20 to -0.14) | 0.00  (-0.01 to 0.01) | -1.5 | 0.00  (-0.02 to 0.01) | -1.2 |
| 6 years | -0.26  (-0.28 to -0.23) | -0.26  (-0.29 to -0.24) | -0.26  (-0.29 to -0.23) | 0.00  (-0.01 to 0.00) | -1.9 | 0.00  (-0.01 to 0.02) | 0.7 |
| 8 years | -0.33  (-0.36 to -0.30) | -0.33  (-0.36 to -0.31) | -0.32  (-0.36 to -0.29) | 0.00  (-0.01 to 0.01) | -0.8 | 0.01  (-0.02 to 0.03) | 2.5 |
| 10 years | -0.47  (-0.50 to -0.44) | -0.47  (-0.50 to -0.44) | -0.46  (-0.50 to -0.43) | 0.00  (-0.01 to 0.01) | 0.4 | 0.01  (-0.01 to 0.03) | 1.6 |
| 12 years | -0.55  (-0.59 to -0.51) | -0.55  (-0.59 to -0.51) | -0.54  (-0.61 to -0.48) | 0.00  (-0.01 to 0.01) | -0.1 | 0.01  (-0.05 to 0.06) | 1.3 |

Estimated mean memory change from baseline was calculated by subtracting the mean baseline memory score from the estimated mean memory score at each study wave. Estimates were obtained adjusting for baseline covariates (age at baseline, race and ethnicity, educational attainment, whether born in southern states, and personality traits), time-varying covariates (household wealth per person, employment status, social contact [marital status, participation in social activities, and contact with children, other family members, or friends], smoking status, frequency of vigorous physical activity, self-reported diagnosed comorbidities [diabetes, hypertension, and stroke], self-rated health, self-reported pain, depressive symptoms measured by CES-D score, and activities of daily living limitations), and memory function in previous waves. Estimates for the sustained loneliness intervention are not shown for 2 years since the baseline because they correspond to the estimates for the baseline loneliness intervention. Some values for percent reduction rounded to 0.00 while still producing a small non-zero percent reduction. Sampling weights for community-dwelling adults in 2006 were used.

Abbreviations: CI = confidence interval; NA = not applicable.

# **Table S9. Estimated mean change in memory scores from baseline under natural course and hypothetical loneliness interventions, stratified by social isolation status at baseline**

| **Years since baseline** | **Mean memory change from baseline** | | | **Impact of interventions** | | | |
| --- | --- | --- | --- | --- | --- | --- | --- |
|  |  |  |  | **Baseline loneliness intervention vs. natural course** | | **Sustained loneliness intervention vs. natural course** | |
|  | **Natural course**  **[A]**  **(95% CI)** | **Baseline loneliness intervention**  **[B]**  **(95% CI)** | **Sustained loneliness intervention**  **[C]**  **(95% CI)** | **Difference**  **[B] - [A]**  **(95% CI)** | **Percentage reduction**  **100***  **([A] - [B])/[A]** | **Difference**  **[C] - [A]**  **(95% CI)** | **Percentage reduction**  **100***  **([A] - [C])/[A]** |
| **Without social isolation** | | | | | | | |
| 2 years | -0.06  (-0.08 to -0.05) | -0.07  (-0.08 to -0.05) | NA | 0.00  (-0.01 to 0.00) | -2.0 | NA | NA |
| 4 years | -0.18  (-0.20 to -0.15) | -0.18  (-0.20 to -0.15) | -0.17  (-0.19 to -0.15) | 0.00  (-0.01 to 0.01) | 0.1 | 0.00  (0.00 to 0.01) | 1.9 |
| 6 years | -0.26  (-0.28 to -0.24) | -0.26  (-0.28 to -0.24) | -0.25  (-0.28 to -0.23) | 0.00  (-0.01 to 0.01) | 0.3 | 0.01  (-0.01 to 0.02) | 3.4 |
| 8 years | -0.34  (-0.37 to -0.32) | -0.34  (-0.37 to -0.32) | -0.34  (-0.36 to -0.31) | 0.00  (-0.01 to 0.01) | 0.2 | 0.01  (-0.01 to 0.03) | 2.4 |
| 10 years | -0.49  (-0.52 to -0.47) | -0.50  (-0.52 to -0.47) | -0.49  (-0.52 to -0.45) | 0.00  (-0.01 to 0.01) | -0.3 | 0.01  (-0.02 to 0.03) | 1.3 |
| 12 years | -0.57  (-0.60 to -0.55) | -0.57  (-0.60 to -0.55) | -0.56  (-0.60 to -0.51) | 0.00  (-0.01 to 0.01) | -0.2 | 0.01  (-0.02 to 0.05) | 2.3 |
| **With social isolation** | | | | | | | |
| 2 years | -0.06  (-0.09 to -0.03) | -0.06  (-0.10 to -0.02) | NA | 0.00  (-0.02 to 0.02) | -5.1 | NA | NA |
| 4 years | -0.18  (-0.22 to -0.14) | -0.18  (-0.23 to -0.14) | -0.18  (-0.23 to -0.14) | 0.00  (-0.02 to 0.01) | -2.6 | 0.00  (-0.02 to 0.02) | -1.6 |
| 6 years | -0.28  (-0.32 to -0.24) | -0.29  (-0.33 to -0.24) | -0.29  (-0.34 to -0.24) | -0.01  (-0.03 to 0.02) | -1.8 | 0.00  (-0.03 to 0.03) | -1.7 |
| 8 years | -0.38  (-0.43 to -0.33) | -0.39  (-0.44 to -0.33) | -0.37  (-0.44 to -0.30) | -0.01  (-0.02 to 0.01) | -1.5 | 0.01  (-0.02 to 0.05) | 3.4 |
| 10 years | -0.53  (-0.58 to -0.48) | -0.53  (-0.59 to -0.47) | -0.51  (-0.58 to -0.44) | 0.00  (-0.02 to 0.02) | 0.0 | 0.02  (-0.02 to 0.06) | 3.8 |
| 12 years | -0.61  (-0.66 to -0.55) | -0.61  (-0.68 to -0.55) | -0.57  (-0.70 to -0.44) | -0.01  (-0.03 to 0.02) | -0.9 | 0.04  (-0.08 to 0.16) | 5.8 |

Estimated mean memory change from baseline was calculated by subtracting the mean baseline memory score from the estimated mean memory score at each study wave. Individuals were defined as socially isolated if they met at least two of the following five conditions: not married or partnered, less than monthly participation in social activities, and less than monthly contact with children, other family members, or friends.

Estimates were obtained adjusting for baseline covariates (age at baseline, sex/gender, race and ethnicity, educational attainment, whether born in southern states, and personality trait), time-varying covariates (household wealth per person, employment status, social contact [marital status, participation in social activities, and contact with children, other family members, or friends], smoking status, frequency of vigorous physical activity, self-reported diagnosed comorbidities [diabetes, hypertension, and stroke], self-rated health, self-reported pain, depressive symptoms measured by CES-D score, and activities of daily living limitations), and memory function in previous waves. Estimates for the sustained loneliness intervention are not shown for 2 years since the baseline because they correspond to the estimates for the baseline loneliness intervention. Some values for percent reduction rounded to 0.00 while still producing a small non-zero percent reduction. Sampling weights for community-dwelling adults in 2006 were used.

Abbreviations: CI = confidence interval; NA = not applicable.

# **Table S10. Estimated mean change in memory scores from baseline under natural course and hypothetical loneliness interventions, using the 3-item UCLA loneliness scale**

| **Years since baseline** | **Mean memory change from baseline** | | | **Impact of interventions** | | | |
| --- | --- | --- | --- | --- | --- | --- | --- |
|  |  |  |  | **Baseline loneliness intervention vs. natural course** | | **Sustained loneliness intervention vs. natural course** | |
|  | **Natural course**  **[A]**  **(95% CI)** | **Baseline loneliness intervention**  **[B]**  **(95% CI)** | **Sustained loneliness intervention**  **[C]**  **(95% CI)** | **Difference**  **[B] - [A]**  **(95% CI)** | **Percentage reduction**  **100***  **([A] - [B])/[A]** | **Difference**  **[C] - [A]**  **(95% CI)** | **Percentage reduction**  **100***  **([A] - [C])/[A]** |
| 4 years | -0.19  (-0.21 to -0.17) | -0.19  (-0.22 to -0.17) | NA | 0.00  (-0.02 to 0.02) | -1.5 | NA | NA |
| 8 years | -0.41  (-0.44 to -0.39) | -0.41  (-0.44 to -0.38) | -0.40  (-0.44 to -0.35) | 0.01  (-0.02 to 0.03) | 1.3 | 0.02  (-0.02 to 0.06) | 4.2 |
| 12 years | -0.64  (-0.67 to -0.60) | -0.63  (-0.68 to -0.59) | -0.61  (-0.68 to -0.53) | 0.00  (-0.02 to 0.03) | 0.3 | 0.03  (-0.03 to 0.09) | 4.8 |

Estimated mean memory change from baseline was calculated by subtracting the mean baseline memory score from the estimated mean memory score at each study wave. Estimates were obtained adjusting for baseline covariates (age at baseline, sex/gender, race and ethnicity, educational attainment, whether born in southern states, and personality traits), time-varying covariates (household wealth per person, employment status, social contact [marital status, participation in social activities, and contact with children, other family members, or friends], smoking status, frequency of vigorous physical activity, self-reported diagnosed comorbidities [diabetes, hypertension, and stroke], self-rated health, self-reported pain, depressive symptoms measured by CES-D score, and activities of daily living limitations), and memory function in previous waves. Estimates for the sustained loneliness intervention are not shown for 2 years since the baseline because they correspond to the estimates for the baseline loneliness intervention. Some values for percent reduction rounded to 0.00 while still producing a small non-zero percent reduction. Sampling weights for community-dwelling adults in 2006 were used.

Abbreviations: CI = confidence interval; NA = not applicable.

# **Table S11. Estimated mean change in memory scores from baseline under natural course and hypothetical loneliness interventions, estimated with inverse probability of censoring weighting for community-dwelling HRS participants at 2006 (N = 17,105)**

| **Years since baseline** | **Mean memory change from baseline** | | | **Impact of interventions** | | | |
| --- | --- | --- | --- | --- | --- | --- | --- |
|  |  |  |  | **Baseline loneliness intervention vs. natural course** | | **Sustained loneliness intervention vs. natural course** | |
|  | **Natural course**  **[A]**  **(95% CI)** | **Baseline loneliness intervention**  **[B]**  **(95% CI)** | **Sustained loneliness intervention**  **[C]**  **(95% CI)** | **Difference**  **[B] - [A]**  **(95% CI)** | **Percentage reduction**  **100***  **([A] - [B])/[A]** | **Difference**  **[C] - [A]**  **(95% CI)** | **Percentage reduction**  **100***  **([A] - [C])/[A]** |
| 2 years | -0.11  (-0.13 to -0.09) | -0.12  (-0.14 to -0.10) | NA | -0.01  (-0.01 to 0.00) | -5.6 | NA | NA |
| 4 years | -0.30  (-0.32 to -0.28) | -0.31  (-0.33 to -0.29) | -0.31  (-0.33 to -0.29) | -0.01  (-0.01 to 0.00) | -1.9 | 0.00  (-0.01 to 0.01) | -1.4 |
| 6 years | -0.44  (-0.46 to -0.42) | -0.44  (-0.47 to -0.42) | -0.43  (-0.46 to -0.41) | 0.00  (-0.01 to 0.01) | -0.6 | 0.01  (-0.01 to 0.02) | 1.4 |
| 8 years | -0.57  (-0.60 to -0.55) | -0.57  (-0.59 to -0.54) | -0.55  (-0.59 to -0.52) | 0.00  (-0.01 to 0.01) | 0.5 | 0.02  (-0.01 to 0.04) | 3.1 |
| 10 years | -0.77  (-0.80 to -0.74) | -0.76  (-0.79 to -0.73) | -0.74  (-0.78 to -0.70) | 0.01  (0.00 to 0.03) | 1.6 | 0.03  (0.00 to 0.06) | 3.8 |
| 12 years | -0.85  (-0.88 to -0.82) | -0.83  (-0.86 to -0.80) | -0.81  (-0.86 to -0.76) | 0.02 (0.00 to 0.03) | 2.0 | 0.04  (-0.01 to 0.09) | 4.5 |

Estimated mean memory change from baseline was calculated by subtracting the mean baseline memory score from the estimated mean memory score at each study wave. Estimates were obtained adjusting for baseline covariates (age at baseline, sex/gender, race and ethnicity, educational attainment, whether born in southern states, and personality traits), time-varying covariates (household wealth per person, employment status, social contact [marital status, participation in social activities, and contact with children, other family members, or friends], smoking status, frequency of vigorous physical activity, self-reported diagnosed comorbidities [diabetes, hypertension, and stroke], self-rated health, self-reported pain, depressive symptoms measured by CES-D score, and activities of daily living limitations), and memory function in previous waves. Inverse probability of censoring was estimated with the same adjustment variables. Estimates for the sustained loneliness intervention are not shown for 2 years since the baseline because they correspond to the estimates for the baseline loneliness intervention. Some values for percent reduction rounded to 0.00 while still producing a small non-zero percent reduction. Sampling weights for community-dwelling adults in 2006 were used.

Abbreviations: CI = confidence interval; NA = not applicable.

# **Table S12. Estimated mean change in memory scores from baseline under natural course and hypothetical loneliness interventions, without applying sampling weights**

| **Years since baseline** | **Mean memory change from baseline** | | | **Impact of interventions** | | | |
| --- | --- | --- | --- | --- | --- | --- | --- |
|  |  |  |  | **Baseline loneliness intervention vs. natural course** | | **Sustained loneliness intervention vs. natural course** | |
|  | **Natural course**  **[A]**  **(95% CI)** | **Baseline loneliness intervention**  **[B]**  **(95% CI)** | **Sustained loneliness intervention**  **[C]**  **(95% CI)** | **Difference**  **[B] - [A]**  **(95% CI)** | **Percentage reduction**  **100***  **([A] - [B])/[A]** | **Difference**  **[C] - [A]**  **(95% CI)** | **Percentage reduction**  **100***  **([A] - [C])/[A]** |
| 2 years | -0.07  (-0.09 to -0.06) | -0.07  (-0.09 to -0.06) | NA | 0.00  (-0.01 to 0.00) | -2.2 | NA | NA |
| 4 years | -0.21  (-0.22 to -0.19) | -0.21  (-0.22 to -0.19) | -0.20  (-0.22 to -0.19) | 0.00  (-0.01 to 0.00) | -0.1 | 0.00  (0.00 to 0.01) | 1.6 |
| 6 years | -0.31  (-0.33 to -0.30) | -0.31  (-0.33 to -0.29) | -0.30  (-0.32 to -0.28) | 0.00  (0.00 to 0.01) | 0.5 | 0.01  (0.00 to 0.02) | 2.9 |
| 8 years | -0.42  (-0.44 to -0.40) | -0.42  (-0.44 to -0.40) | -0.41  (-0.43 to -0.38) | 0.00  (0.00 to 0.01) | 0.4 | 0.01  (0.00 to 0.03) | 3.0 |
| 10 years | -0.59  (-0.61 to -0.57) | -0.59  (-0.61 to -0.57) | -0.57  (-0.60 to -0.55) | 0.00  (-0.01 to 0.01) | -0.1 | 0.02  (0.00 to 0.04) | 3.0 |
| 12 years | -0.68  (-0.70 to -0.65) | -0.68  (-0.71 to -0.65) | -0.65  (-0.68 to -0.61) | 0.00  (-0.01 to 0.01) | -0.1 | 0.03 (0.00 to 0.06) | 4.7 |

Estimated mean memory change from baseline was calculated by subtracting the mean baseline memory score from the estimated mean memory score at each study wave. Estimates were obtained adjusting for baseline covariates (age at baseline, sex/gender, race and ethnicity, educational attainment, whether born in southern states, and personality traits), time-varying covariates (household wealth per person, employment status, social contact [marital status, participation in social activities, and contact with children, other family members, or friends], smoking status, frequency of vigorous physical activity, self-reported diagnosed comorbidities [diabetes, hypertension, and stroke], self-rated health, self-reported pain, depressive symptoms measured by CES-D score, and activities of daily living limitations), and memory function in previous waves. Estimates for the sustained loneliness intervention are not shown for 2 years since the baseline because they correspond to the estimates for the baseline loneliness intervention. Some values for percent reduction rounded to 0.00 while still producing a small non-zero percent reduction.

Abbreviations: CI = confidence interval; NA = not applicable.

# **Table S13. Estimated mean change in memory scores from baseline under natural course and hypothetical loneliness interventions, after limiting the sample to only one participant within a household (N = 7,627)**

| **Years since baseline** | **Mean memory change from baseline** | | | **Impact of interventions** | | | |
| --- | --- | --- | --- | --- | --- | --- | --- |
|  |  |  |  | **Baseline loneliness intervention vs. natural course** | | **Sustained loneliness intervention vs. natural course** | |
|  | **Natural course**  **[A]**  **(95% CI)** | **Baseline loneliness intervention**  **[B]**  **(95% CI)** | **Sustained loneliness intervention**  **[C]**  **(95% CI)** | **Difference**  **[B] - [A]**  **(95% CI)** | **Percentage reduction**  **100***  **([A] - [B])/[A]** | **Difference**  **[C] - [A]**  **(95% CI)** | **Percentage reduction**  **100***  **([A] - [C])/[A]** |
| 2 years | -0.07  (-0.08 to -0.05) | -0.07  (-0.09 to -0.05) | NA | 0.00  (-0.01 to 0.00) | -4.4 | NA | NA |
| 4 years | -0.19  (-0.21 to -0.17) | -0.19  (-0.21 to -0.18) | -0.19  (-0.21 to -0.17) | 0.00  (-0.01 to 0.00) | -1.7 | 0.00  (-0.01 to 0.01) | 0.1 |
| 6 years | -0.29  (-0.31 to -0.27) | -0.29  (-0.31 to -0.27) | -0.29  (-0.31 to -0.26) | 0.00  (-0.01 to 0.01) | -0.6 | 0.00  (-0.01 to 0.02) | 1.6 |
| 8 years | -0.39  (-0.41 to -0.37) | -0.39  (-0.41 to -0.37) | -0.38  (-0.41 to -0.35) | 0.00  (-0.01 to 0.01) | -0.1 | 0.01  (-0.01 to 0.03) | 2.7 |
| 10 years | -0.55  (-0.57 to -0.52) | -0.55  (-0.58 to -0.52) | -0.53  (-0.57 to -0.49) | 0.00  (-0.01 to 0.01) | -0.6 | 0.02  (-0.01 to 0.04) | 2.7 |
| 12 years | -0.62  (-0.65 to -0.59) | -0.63  (-0.66 to -0.60) | -0.60  (-0.66 to -0.55) | 0.00  (-0.01 to 0.01) | -0.6 | 0.02  (-0.03 to 0.07) | 3.3 |

Estimated mean memory change from baseline was calculated by subtracting the mean baseline memory score from the estimated mean memory score at each study wave. Estimates were obtained adjusting for baseline covariates (age at baseline, sex/gender, race and ethnicity, educational attainment, whether born in southern states, and personality traits), time-varying covariates (household wealth per person, employment status, social contact [marital status, participation in social activities, and contact with children, other family members, or friends], smoking status, frequency of vigorous physical activity, self-reported diagnosed comorbidities [diabetes, hypertension, and stroke], self-rated health, self-reported pain, depressive symptoms measured by CES-D score, and activities of daily living limitations), and memory function in previous waves. Estimates for the sustained loneliness intervention are not shown for 2 years since the baseline because they correspond to the estimates for the baseline loneliness intervention. Some values for percent reduction rounded to 0.00 while still producing a small non-zero percent reduction. Sampling weights for community-dwelling adults in 2006 were used.

Abbreviations: CI = confidence interval; NA = not applicable.

# **Table S14. Estimated mean change in memory scores from baseline under natural course and hypothetical loneliness interventions, estimated with ten imputed datasets**

| **Years since baseline** | **Mean memory change from baseline** | | | **Impact of interventions** | | | |
| --- | --- | --- | --- | --- | --- | --- | --- |
|  |  |  |  | **Baseline loneliness intervention vs. natural course** | | **Sustained loneliness intervention vs. natural course** | |
|  | **Natural course**  **[A]**  **(95% CI)** | **Baseline loneliness intervention**  **[B]**  **(95% CI)** | **Sustained loneliness intervention**  **[C]**  **(95% CI)** | **Difference**  **[B] - [A]**  **(95% CI)** | **Percentage reduction**  **100***  **([A] - [B])/[A]** | **Difference**  **[C] - [A]**  **(95% CI)** | **Percentage reduction**  **100***  **([A] - [C])/[A]** |
| 2 years | -0.06  (-0.08 to -0.05) | -0.06  (-0.08 to -0.05) | NA | 0.00  (-0.01 to 0.01) | 0.2 | NA | NA |
| 4 years | -0.18  (-0.19 to -0.16) | -0.18  (-0.19 to -0.16) | -0.18  (-0.19 to -0.16) | 0.00  (-0.01 to 0.01) | -0.3 | 0.00  (0.00 to 0.01) | 1.8 |
| 6 years | -0.27  (-0.29 to -0.25) | -0.27  (-0.28 to -0.25) | -0.26  (-0.28 to -0.24) | 0.00  (-0.01 to 0.01) | 0.3 | 0.01  (0.00 to 0.02) | 2.8 |
| 8 years | -0.36  (-0.38 to -0.34) | -0.36  (-0.38 to -0.34) | -0.35  (-0.37 to -0.33) | 0.00  (-0.01 to 0.01) | -0.1 | 0.01  (0.00 to 0.03) | 2.9 |
| 10 years | -0.51  (-0.53 to -0.48) | -0.51  (-0.53 to -0.48) | -0.49  (-0.52 to -0.46) | 0.00  (-0.01 to 0.01) | 0.0 | 0.02  (-0.01 to 0.04) | 3.0 |
| 12 years | -0.58  (-0.61 to -0.56) | -0.58  (-0.61 to -0.56) | -0.56  (-0.60 to -0.52) | 0.00  (-0.01 to 0.01) | -0.2 | 0.02  (-0.02 to 0.05) | 2.9 |

Estimated mean memory change from baseline was calculated by subtracting the mean baseline memory score from the estimated mean memory score at each study wave. Estimates were obtained adjusting for baseline covariates (age at baseline, sex/gender, race and ethnicity, educational attainment, whether born in southern states, and personality traits), time-varying covariates (household wealth per person, employment status, social contact [marital status, participation in social activities, and contact with children, other family members, or friends], smoking status, frequency of vigorous physical activity, self-reported diagnosed comorbidities [diabetes, hypertension, and stroke], self-rated health, self-reported pain, depressive symptoms measured by CES-D score, and activities of daily living limitations), and memory function in previous waves. Estimates for the sustained loneliness intervention are not shown for 2 years since the baseline because they correspond to the estimates for the baseline loneliness intervention. Some values for percent reduction rounded to 0.00 while still producing a small non-zero percent reduction. Sampling weights for community-dwelling adults in 2006 were used.

Abbreviations: CI = confidence interval; NA = not applicable.

# **Table S15. Observed mean memory change over follow-up and mean memory change over follow-up estimated from TMLE under the natural course**

| **Years since baseline** | **Observed mean memory change from baseline**  **(95% CI)** | **Mean memory change predicted from baseline estimated from TMLE under the natural course**  **(95% CI)** |
| --- | --- | --- |
| 2 | -0.06  (-0.08 to -0.05) | -0.06  (-0.08 to -0.05) |
| 4 | -0.18  (-0.19 to -0.16) | -0.18  (-0.19 to -0.16) |
| 6 | -0.27  (-0.28 to -0.25) | -0.27  (-0.29 to -0.25) |
| 8 | -0.36  (-0.37 to -0.34) | -0.36  (-0.38 to -0.34) |
| 10 | -0.50  (-0.53 to -0.48) | -0.51  (-0.53 to -0.49) |
| 12 | -0.58  (-0.60 to -0.56) | -0.58  (-0.61 to -0.56) |

Estimated mean memory change from baseline was calculated by subtracting the mean baseline memory score from the estimated mean memory score at each study wave. Sampling weights for community-dwelling adults in 2006 were used.

Abbreviations: CI = confidence interval; TMLE = targeted maximum likelihood estimation. **Reference**

1. Howard VJ, Woolson RF, Egan BM, Nicholas JS, Adams RJ, Howard G, et al. Prevalence of hypertension by duration and age at exposure to the stroke belt. J Am Soc Hypertens. 2010;4(1):32-41.

2. Glymour MM, Kosheleva A, Boden-Albala B. Birth and adult residence in the Stroke Belt independently predict stroke mortality. Neurology. 2009;73(22):1858-65.

3. Gilsanz P, Mayeda ER, Glymour MM, Quesenberry CP, Whitmer RA. Association Between Birth in a High Stroke Mortality State, Race, and Risk of Dementia. JAMA Neurol. 2017;74(9):1056-62.

4. Buecker S, Maes M, Denissen JJ, Luhmann M. Loneliness and the Big Five personality traits: A meta–analysis. European Journal of Personality. 2020;34(1):8-28.

5. Sutin AR, Stephan Y, Luchetti M, Terracciano A. Five-factor model personality traits and cognitive function in five domains in older adulthood. BMC Geriatr. 2019;19(1):343.

6. Yin J, Lassale C, Steptoe A, Cadar D. Exploring the bidirectional associations between loneliness and cognitive functioning over 10 years: the English longitudinal study of ageing. Int J Epidemiol. 2019;48(6):1937-48.

7. Okely JA, Deary IJ. Longitudinal Associations Between Loneliness and Cognitive Ability in the Lothian Birth Cohort 1936. The journals of gerontology Series B, Psychological sciences and social sciences. 2019;74(8):1376-86.

8. Zhong BL, Chen SL, Tu X, Conwell Y. Loneliness and Cognitive Function in Older Adults: Findings From the Chinese Longitudinal Healthy Longevity Survey. The journals of gerontology Series B, Psychological sciences and social sciences. 2017;72(1):120-8.

9. Hawkley LC, Cacioppo JT. Loneliness matters: a theoretical and empirical review of consequences and mechanisms. Ann Behav Med. 2010;40(2):218-27.

10. Hawkley LC, Thisted RA, Cacioppo JT. Loneliness predicts reduced physical activity: cross-sectional & longitudinal analyses. Health Psychol. 2009;28(3):354-63.

11. DeWall CN, Pond Jr RS. Loneliness and smoking: The costs of the desire to reconnect. Self and Identity. 2011;10(3):375-85.

12. Iso-Markku P, Aaltonen S, Kujala UM, Halme HL, Phipps D, Knittle K, et al. Physical Activity and Cognitive Decline Among Older Adults: A Systematic Review and Meta-Analysis. JAMA Netw Open. 2024;7(2):e2354285.

13. Reitz C, Luchsinger J, Tang MX, Mayeux R. Effect of smoking and time on cognitive function in the elderly without dementia. Neurology. 2005;65(6):870-5.

14. Zeki Al Hazzouri A, Caunca MR, Nobrega JC, Elfassy T, Cheung YK, Alperin N, et al. Greater depressive symptoms, cognition, and markers of brain aging: Northern Manhattan Study. Neurology. 2018;90(23):e2077-e85.

15. Sabia S, Marmot M, Dufouil C, Singh-Manoux A. Smoking history and cognitive function in middle age from the Whitehall II study. Arch Intern Med. 2008;168(11):1165-73.

16. Rehkopf DH, Glymour MM, Osypuk TL. The Consistency Assumption for Causal Inference in Social Epidemiology: When a Rose is Not a Rose. Curr Epidemiol Rep. 2016;3(1):63-71.

17. Hernan MA, Robins JM. Causal Inference: What If2020.

18. Shaw C, Wu Y, Zimmerman SC, Hayes-Larson E, Belin TR, Power MC, et al. Comparison of Imputation Strategies for Incomplete Longitudinal Data in Lifecourse Epidemiology. Am J Epidemiol. 2023.

19. Van Buuren S. Flexible imputation of missing data: CRC press; 2018.

20. van Buuren S, Groothuis-Oudshoorn K. mice: Multivariate Imputation by Chained Equations in R. J Stat Softw. 2011;45(3):1-67.

21. Rubin DB. Multiple imputation for nonresponse in surveys: John Wiley & Sons; 2004.

22. Wu Q, Tchetgen Tchetgen EJ, Osypuk TL, White K, Mujahid M, Maria Glymour M. Combining direct and proxy assessments to reduce attrition bias in a longitudinal study. Alzheimer Dis Assoc Disord. 2013;27(3):207-12.
